# Bacteria From the Multi-Contaminated Tinto River Estuary (SW, Spain) Show High Multi-Resistance to Antibiotics and Point to *Paenibacillus* spp. as Antibiotic-Resistance-Dissemination Players

**DOI:** 10.3389/fmicb.2019.03071

**Published:** 2020-01-10

**Authors:** Benedito Eduardo-Correia, Héctor Morales-Filloy, José P. Abad

**Affiliations:** Department of Molecular Biology, Faculty of Sciences-Biology Building, Universidad Autónoma de Madrid, Madrid, Spain

**Keywords:** antibiotic resistance, estuary, environmental bacteria isolates, multi-drug resistance, *Paenibacillus*, tinto river, resistance dissemination, contaminated environment

## Abstract

Bacterial resistance to antibiotics is an ever-increasing phenomenon that, besides clinical settings, is generally assumed to be prevalent in environmental soils and waters. The analysis of bacteria resistant to each one of 11 antibiotics in waters and sediments of the Huelva’s estuary, a multi-contaminated environment, showed high levels of bacteria resistant mainly to Tm, among others. To further gain knowledge on the fate of multi-drug resistance (MDR) in environmental bacteria, 579 ampicillin-resistant bacteria were isolated tested for resistance to 10 antibiotics. 92.7% of the isolates were resistant to four or more antibiotic classes, indicating a high level of multi-resistance. 143 resistance profiles were found. The isolates with different MDR profiles and/or colony morphologies were phylogenetically ascribed based on 16S rDNA to phyla *Proteobacteria, Firmicutes, Actinobacteria*, and *Bacteroidetes*, including 48 genera. Putative intrinsic resistance was detected in different phylogenetic groups including genera *Altererythrobacter*, *Bacillus*, *Brevundimonas*, *Erythrobacter*, *Mesonia*, *Ochrobactrum*, and *Ponticaulis*. Correlation of the presence of pairs of the non-intrinsic-resistances in phylogenetic groups based on the kappa index (κ) highlighted the co-habitation of some of the tested pairs at different phylogenetic levels. Maximum correlation (κ = 1.000) was found for pairs Cz^R^/Tc^R^ in Betaproteobacteria, and Cc^R^/Tc^R^ and Em^R^/Sm^R^ in Sphingobacteriia at the class level, while at the genus level, was found for Cc^R^/Tc^R^ and Nx^R^/Tm^R^ in *Mesonia*, Cz^R^/Tm^R^ and Em^R^/Km^R^ in *Paenibacillus*, and Cc^R^/Em^R^ and Rp^R^/Tc^R^ in *Pseudomonas*. These results could suggest the existence of intra-class and intra-genus-transmissible genetic elements containing determinants for both members of each pair. Network analysis based on κ values higher than 0.4 indicated the sharing of paired resistances among several genera, many of them centered on the *Paenibacillus* node and raising the hypothesis of inter-genera transmission of resistances interconnected through members of this genus. This is the first time that a possible hotspot of resistance interchange in a particular environment may have been detected, opening up the possibility that one, or a few, bacterial members of the community could be important promoters of antibiotic resistance (AR) dissemination in this environment’s bacterial population. Further studies using the available isolates will likely give insights of the possible mechanisms and genetic elements involved.

## Introduction

The antibiotic resistance (AR) of bacterial pathogens is currently a worldwide problem with severe consequences for the treatment of infectious diseases (World Health Organization [WHO], 2018). However, AR existed in prehistoric (Perry et al., 2016) and more recent times (Devault et al., 2017); and its origin is not directly related to the clinical use of antibiotics. It was probably triggered by the appearance of antibiotics in the environment when their producers needed to be protected from their effects (Jiang et al., 2017). The environmental role of these compounds is not yet clear and for a long time was considered only ecological. This function was demonstrated only in a few cases (Currie et al., 1999; Neeno-Eckwall et al., 2001; Haas and Défago, 2005). Antibiotic concentrations in nature may not be enough for this purpose (Fajardo et al., 2009), while low concentrations have pleiotropic effects that are not related to competition (Davies et al., 2006; Linares et al., 2006; Yim et al., 2007; Fajardo and Martinez, 2008; Sengupta et al., 2013; Andersson and Hughes, 2014; Goneau et al., 2015; Martínez, 2017; Xiong et al., 2017). However, low individual concentrations of a cocktail of antibiotics could have a combined effect [reviewed by Danner et al. (2019)]. Additionally, sub-lethal concentrations select resistant phenotypes from mutations or horizontal gene transfer (HGT) that sometimes use mobile genetic elements (MGEs) (Kohanski et al., 2010; Baharoglu and Mazel, 2011; Gutierrez et al., 2013; Haaber et al., 2017; Lekunberri et al., 2017). The AR in the environment is a natural process but is also promoted by anthropogenic pollutants like antibiotics, biocides, heavy metals, hydrocarbons, pesticides, and nanomaterials, among others (Baker-Austin et al., 2006; Dealtry et al., 2014; Pal et al., 2015, 2017; Chen et al., 2017; Poole, 2017; Wang et al., 2018; Nguyen et al., 2019). Therefore, naturally antibiotic-resistant bacteria (ARB) could be a prime source for antibiotic resistance genes (ARGs) found in pathogens as certain findings may indicate (Rodríguez et al., 2004; Poirel et al., 2005, 2012; Yang J. et al., 2013; Jiang et al., 2017), but may later have evolved and be selected.

The interaction of microorganisms from natural, clinical, and farming sources could select multi-resistant bacteria (MRB), severely affecting environments, change biodiversity, and modify evolution paths in favor of resistants (Ding and He, 2010; Gupta, 2011; Proia et al., 2013; Laverman et al., 2015; Hiltunen et al., 2017; Grenni et al., 2018). The major challenge posed by ARB is their acquisition of MDR. Several mechanisms for the accumulation of resistances are known (Cambray et al., 2010; Michael et al., 2012; Brown-Jaque et al., 2015; Carraro et al., 2016, 2017; Ramsay and Firth, 2017). However, MDR spreads in the environment and the resistance patterns that are passed-on together or transferred to pathogenic bacteria, or how these bacteria can model their environments remain unknown (Chamosa et al., 2017). Studies to estimate the risk of HGT between environmental and pathogenic bacteria have identified hotspots of transfer including wastewater treatment plants (WWTPs), which are recognized as one of the main sources of antibiotics pollution in surface waters (Guo et al., 2017; Manaia et al., 2018). Few experimental studies on the specific transmissible elements and their dissemination capabilities have been conducted. The structure, function, and inter-relationships of the environmental microbiomes are complex phenomena only partially studied and their comprehension needs to use multidisciplinary approaches and methodologies, including different culture-dependent and independent approaches, to analyze in a “one health” approach how the AR affects environmental microbial biodiversity and limits the use of antibiotics (Stefani et al., 2015; Ellington et al., 2017; Hiltunen et al., 2017; Tripathi and Cytryn, 2017).

Recent studies have shown that many rivers worldwide contain relevant concentrations of antibiotics (Boxall and Wilkinson, 2019; Wilkinson and Boxall, 2019) up to 50 μg/L in some African countries or up to 10 μg/L in European ones (Danner et al., 2019). The prescriptions of antibiotics in Spain has risen from 2012 to 2018 (European Centre for Disease Prevention and Control [ECDC], 2017)^1^ and several studies on the concentration of antibiotics in Spain’s surface waters have shown concentrations of 1.3 μg/L on average [reviewed in Danner et al. (2019)]. Running surface waters such as rivers and their estuaries in anthropogenically influenced areas are a means for antibiotic resistance dissemination through their courses and receiving waters. Some rivers receive effluents from WWTPs facilities, which are not able in most cases to completely eliminate pharmaceuticals such as antibiotics, ARBs, or ARGs, even with the implementation of disinfection protocols (Loos et al., 2012; Suzuki et al., 2017; Barancheshme and Munir, 2018; Ju et al., 2019). So, these bacteria and chemicals carried by the treated wastewater can affect the environment by selection of antibiotic resistant bacteria, changing biodiversity and increasing the levels of resistant and MRB (Sanseverino et al., 2018). Also soils and sediments can receive antibiotics and other pollutants that are not completely degraded and can impact their microbial activity and diversity (Cycon et al., 2019). In addition, other substances can be contaminating rivers and estuaries when they receive effluents from industries established within their borders.

**FIGURE 1:**
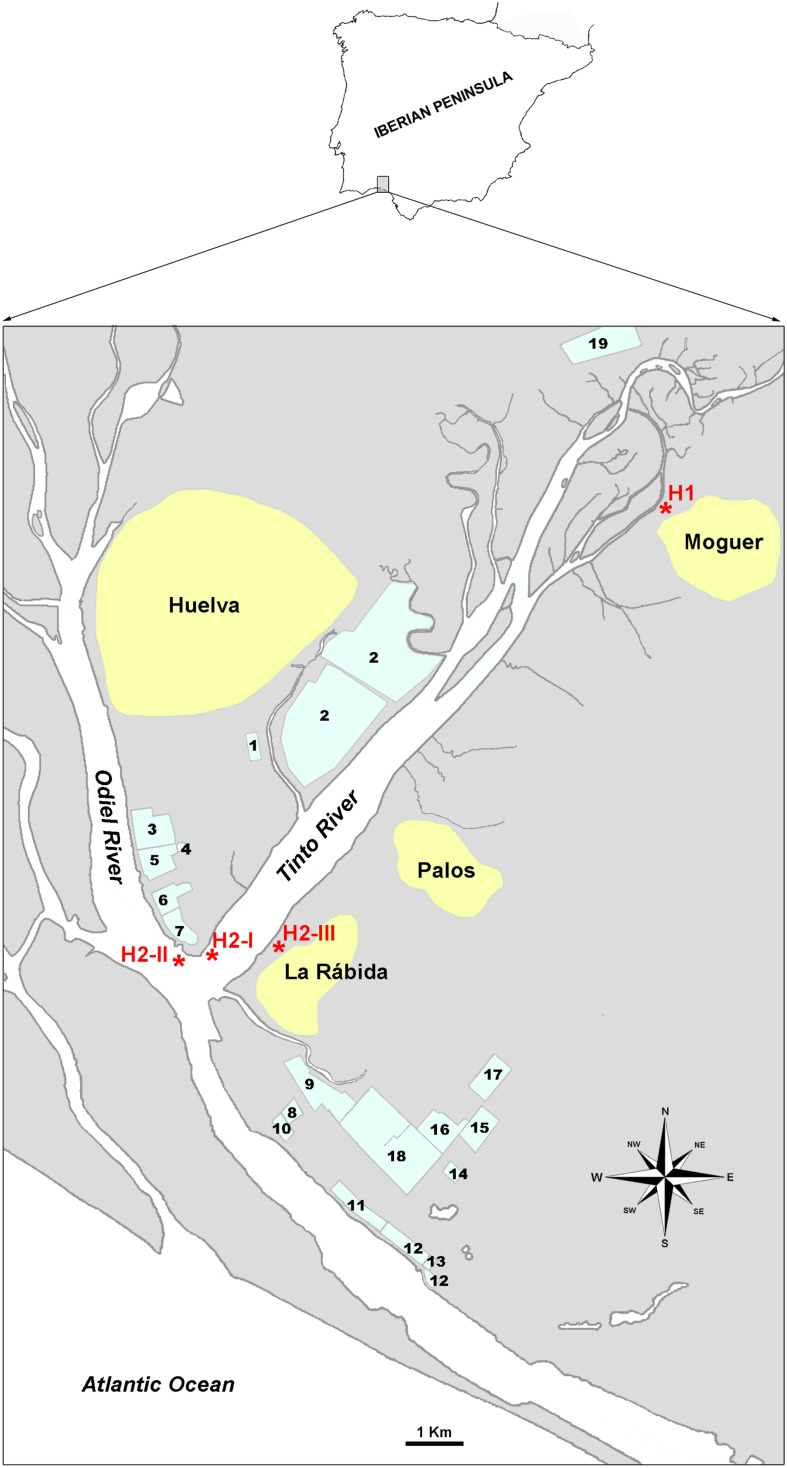
Situation map of sampling sites. Sampling points are labeled as H1 and H2-I, H2-II and H2-III. 1. WWTP of Huelva. 2. Phosphogypsum deposits. 3., 6., and 15. Fertilizers plants. 4. Liquefied gases plant. 5. Copper metallurgy plant. 7. Thermic energy plant. 8. Chemicals production plant. 9. Hydrocarbons storage. 10. Petroleum coque storage. 11. Natural gas plant. 12. Petrochemical products storage. 13. Palm oil refinery. 14. Biodiesel plant. Pharmaceuticals and food supplements plant. 17. Pigments plant. 18. Oil refinery. 19. Biomass thermic plant (old cellulose plant).

The Huelva estuary (SW, Spain) receives waters from two acidic and heavy metal-rich rivers that mix with seawater from the Atlantic Ocean. The WWTP of Huelva, a city with about 145,000 inhabitants, processes urban wastewaters about 240.000 population equivalents^2^; and emits its effluents into the estuary. Also, effluents from a number of industries around the estuary, including oil refineries, metallurgic industries, fertilizers plants, etc., (Figure 1) arrive in the estuary to create a multi-contaminated environment (Davis et al., 2000; Sainz et al., 2004; Pérez-López et al., 2011; Amils, 2016; Papaslioti et al., 2018). Moreover, some studies have reported the presence of radioactivity in the estuary that is related to the fertilizer production that uses uranium-containing phosphogypsum, which accumulates in sediments (Martínez-Aguirre and García-León, 1997; Elbaz-Poulichet et al., 1999). As stated above, several studies have reported that the appearance of antibiotic resistance can be facilitated by the presence in the environment of heavy metals that are abundant in the Huelva estuary; hydrocarbons that come from oil refineries’ management or accidental spills; and for antibiotics that are likely provided by the WWTP. Due to the prevalence of and magnitude of the metals pollution, previous studies have been limited to this aspect.

This study reports our conclusions as to the prevalence of culturable ARB in the surface water and sediments of the Huelva estuary with an aim to contribute to the current knowledge on the diversity and fate of MDR of culturable environmental bacteria as well as to collect biological materials for further study on the transmission of antibiotic resistance and the MGEs involved. 579 ampicillin-resistant bacteria were isolated and their MDR levels and resistance profile diversity were determined. The analysis of these data identified putative intrinsic resistances in different phylogenetic groups as well as correlations of the presence of pairs of acquired resistances mainly at the genus level. A network analysis of the best correlations suggests that members of the *Paenibacillus* genus could be important players in the dissemination of resistance in this environment.

## Materials and Methods

### Huelva’s Estuary Sampling

Samples were collected in areas named H1 and H2 (Figure 1). H1 locates at 37° 16′ 57.81′′ N, −6° 50′ 59.68′′ W, and H2 was an aliquot mix from samples taken at: H2-I (37° 12′ 48.92′′ N, −6° 56′15.23′′ W), H2-II (37° 12′ 41.30′′ N, −6° 56′ 27.21′′ W), and H2-III (37° 12′41.51′′ N, −6° 55′49.97′′ W). Surface water (H1L, H2L) and sediments (H1S, H2S) were collected.

### Physicochemical Parameters of Huelva’s Estuary

They were measured *in situ* using the multi-parametric portable sensor Thermo Orion 290A (Thermo Fisher Scientific, United States). The elemental compositions were measured by the Servicio Interdepartamental de Investigación (SIdI) of the Universidad Autónoma de Madrid^3^, using Inductively Coupled Plasma Mass Spectrometry (ICP-MS) for water samples and Total X-Ray Reflection Fluorescence (TXRF) for sediments.

### Culture and Isolation of ARB

In order to quantify total culturable and resistant bacteria, dilutions in the culture media (10^0^–10^–6^) of the liquid samples and culture-media-suspended sediments were inoculated by extension in Petri dishes containing autoclaved solid media. Two culture medias were used: marine [55.1 g/l Difco Marine agar (BD, Sparks, MD, United States)] and nutritive [3 g/l meat extract (Merck, Darmstadt, Germany), 5 g/l NaCl (Merck), 10 g/l bactopeptone (Labs. Conda, Madrid, Spain), 15 g/l agar (Labs. Conda)]. After autoclaving (121°C, 20′, 1 atm), media were left to cool down to 50°C and when needed, supplemented with one out of 11 antibiotics at the following final concentrations: 100 μg/ml for streptomycin (Sm) (Duchefa Biochemie, Haarlem, Netherlands); 50 μg/ml for ampicillin (Ap) and vancomycin (Vm) (Duchefa), ceftazidime (Cz), kanamycin (Km), and nalidixic acid (Nx) (Sigma-Aldrich, St. Louis, MO, United States); 25 μg/ml for chloramphenicol (Cc) and erythromycin (Em) (Duchefa), and tetracycline (Tc) (Sigma); 16 μg/ml for trimethoprim (Tm) (Duchefa); and 8 μg/ml for rifampicin (Rp) (Duchefa). To all the media, cycloheximide (Duchefa) was added at the final concentration of 75 μg/ml. Three dishes per dilution were inoculated and cultured at 30°C for 5 days before the colony counts were done. Bacteria growing on Ap-containing media (Ap^R^) and showing different colony morphologies were selected for isolation. When many colonies with the same morphology appeared from a sample, one out of every three was collected. Isolation from the initial plating colonies was performed by successive passages on solid media containing the same selective agent until isolated colonies of the same morphology were obtained during three consecutive replatings. Isolates were stored at −80°C in 50% (v/v) of glycerol (87%, Merck) and the corresponding liquid culture medium.

Multi-resistance of the isolates was tested on the solid medium used for their isolation containing individual antibiotics at the concentrations indicated above. Dishes inoculated with Ap served as growth control.

### DNA Extraction

DNA was isolated from individual bacterial colonies according to Kai et al. (2006), or using the Ultraclean Microbial DNA Isolation Kit (MOBIO, Carlsbad, CA, United States) according to the manufacturer’s instructions [except for a pretreatment with lysozyme 10 mg/ml (Sigma) for 1 h at 37°C].

### Phylogenetic Analysis

Conventional PCR amplification of 16S rDNA was performed using 27F and 1492R universal primers (Weisburg et al., 1991). Reactions (50 μl) contained: 3 μl DNA sample, 0.5 μM each primer (Sigma), 0.5 μM dNTPs (Invitrogen, Carlsbad, CA, United States), 5 μl 10X buffer (Promega, Fitchburg, WI, United States), 3 mM MgCl_2_ (Promega) and 2.5 U Taq polymerase (Promega). Amplifications [2720 thermocycler (Applied Biosystems, Waltham, MA, United States)] were done using the following program: 94°C 5 min; 30 cycles: 94°C 1 min, 54°C 1 min, 72°C 1.30 min; 72°C 10 min. Amplicons were purified by precipitation by mixing the reaction solution with equal volume of 20% (w/v) PEG [polyethylene glycol 6000 (Merck) in 2.5 M NaCl (Merck)], incubating for 30 min at 37°C with shaking every 10 min, centrifuging at 2850 × *g* for 15 min and washing the pellet twice with 80% (v/v) ethanol (Merck). After vacuum drying the DNA was resuspended in MilliQ water and sequenced by Macrogen Inc., (Seoul, South Korea). Sequences were deposited at the European Molecular Biology Laboratory (EMBL) (Acc. No.: LT601033-LT601378).

Isolates were initially assigned to the genus or species with the highest sequence similarity using NCBI BLAST (Altschul et al., 1990); and were further identified compared with type strains in the phylogenetic trees built with the sequences at the Ribosomal Database Project (RDP) (RRID: SCR_006633) (Cole et al., 2014). Clustal X^4^ was used for alignments and building of neighbor-joining trees, with 1000 repetitions for bootstrapping.

### Statistical Analysis

Data were analyzed using SPSS Statistics V21.0 software (IBM Corporation, New York, NY, United States) and Microsoft Excel 2013. The normality of the distribution of the variables was evaluated by using the Kolmogorov–Smirnov test.

To characterize MDR level the multiple-antibiotic resistance index (MAR) was calculated for each isolate and for the groups of them considered as the median (*x̃*) of the isolates’ MARs (Krumperman, 1983).

The non-parametric tests of Mann-Whitney and Kruskal–Wallis were performed to compare the MARs median. Statistical correlations between the sampling areas or culture media with the observed resistance to each antibiotic were analyzed using contingency tables and contrasting Chi-square.

The kappa indexes (κ) for association of resistance pairs were calculated for groups with eight or more isolates. Networking among pairs with κ0.4 by class and genus were represented using gephi^5^.

For all analysis, results were considered statistically significant for *p* < 0.05.

Distributions of resistants among zones or phylogenetic groups are presented by using the Circos online application^6^.

## Results

### Physicochemical Parameters

Physicochemical parameters of water samples were: pH 6.2–6.5, temperature: 17.5–20.6°C, salinity: 27.1–31.9, conductivity: 42.1–48.6 mS/cm, and redox potential: 159.0–208.0 mV (Supplementary Table S1). Elemental composition of samples is reported in Supplementary Tables S2, S3.

### Abundance of Antibiotic-Resistant Culturable Bacteria

Total culturable bacteria reported as CFU/mL for water and as CFU/g for sediments were, respectively: in marine medium: 1.1 ± 0.042 × 10^6^ and 4.7 ± 2.2 × 10^7^ for H1, and 6.8 ± 1.1 × 10^4^ and 3.0 ± 1.1 × 10^7^ for H2, while in nutritive one: 5.1 ± 0.13 × 10^4^ and 9.6 ± 1.2 × 10^5^ for H1 and 1.4 ± 0.037 × 10^3^ and 1.1 ± 0.4 × 10^5^ for H2.

**FIGURE 2:**
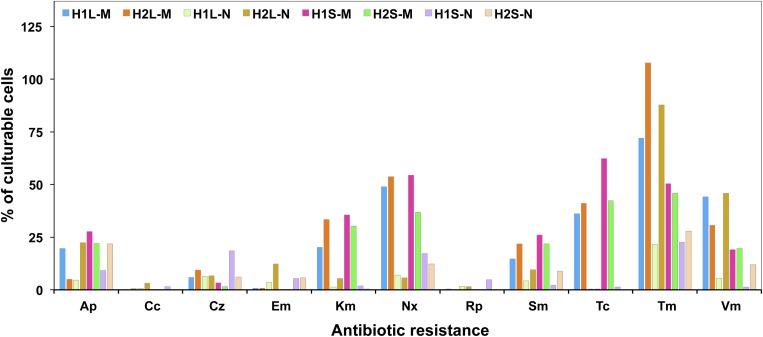
Relative abundance of culturable resistant bacteria in waters and sediments of the sampling sites.

Relative abundances of resistance to each antibiotic (as% of resistants/total culturable bacteria) (Figure 2) were, in general, higher for bacteria tested on the marine rather than on the nutritive medium. The highest was obtained for Tm-resistants, irrespective of the sampling zone or the culture medium. However, Tc-resistants were common among the bacteria grown on marine medium but scarce among those from nutritive one. Percentages of resistants to Cc, Em, or Rp, were low, but higher in the nutritive than in the marine medium.

### Ampicillin-Resistant Bacteria

579 ampicillin-resistant bacteria were isolated corresponding to 286 and 293 colonies from H1 and H2 areas, respectively. From each zone, 70.63 and 74.06%, respectively, were isolates from the marine medium. Table 1 summarizes the distribution of isolates per sample.

**TABLE 1:** Characteristics and distribution of Ap^R^ isolates among the samples tested on nutritive and marine media.

	**Samples**								
	
	**H**			**H1**			**H2**		
			
	**H**	**HL**	**HS**	**H1**	**H1L**	**H1S**	**H2**	**H2L**	**H2S**

**Culture medium**									
***M***									
Median	0.545	0.545	**0.636**	0.545	0.545	**0.636**	0.545	0.545	**0.682**
% MAR≥0.636	45.8	39.7	**64.7**	47.5	42.6	**61.1**	44.2	37.3	**68.8**
N° profiles (% total)	**85 (59.4)**	**75 (52.4)**	34 (23.8)	57 (39.9)	49 (34.3)	25 (17.5)	64 (44.8)	54 (37.8)	22 (15.4)
N° isolates (% total)	419 (72.4)	317 (54.7)	102 (17.6)	202 (34.9)	148 (25.6)	54 (9.3)	217 (37.5)	169 (29.2)	48 (8.3)
Ratio (%) pofiles/isolates	20.3	23.6	33.3	28.2	33.1	46.3	29.5	32.0	45.8
***N***									
Median	**0.727**	0.545	**0.727**	**0.636**	0.545	**0.636**	**0.727**	0.364	**0.727**
% MAR≥0.636	**66.9**	40.4	**79.6**	**58.3**	44.1	**68.0**	**76.3**	33.3	**89.7**
N° profiles (% total)	64 (44.8)	35 (24.5)	43 (30.1)	50 (35.0)	26 (18.2)	35 (24.5)	22 (15.4)	13 (9.1)	11 (7.7)
N° isolates (% total)	160 (27.6)	52 (9.0)	108 (18.7)	84 (14.5)	34 (5.9)	50 (8.6)	76 (13.1)	18 (3.1)	58 (10.0)
Ratio (%) profiles/isolates	40.0	67.3	39.8	59.5	76.5	70.0	28.9	72.2	19.0
**Total**									
Median	**0.636**	0.545	**0.727**	**0.636**	0.545	**0.636**	**0.636**	0.545	**0.727**
% MAR≥0.636	**51.6**	39.8	**72.4**	**50.7**	42.9	**64.4**	**52.6**	36.9	**80.2**
N° profiles (% total)	**143 (100)**	**107 (74.8)**	**73 (51.0)**	**104 (72.7)**	**73 (51.0)**	58 (40.6)	**82 (57.3)**	66 (46.2)	30 (21.0)
N° isolates (% total)	579 (100)	369 (63.7)	210 (36.3)	286 (49.4)	182 (31.4)	104 (18.0)	293 (50.1)	187 (32.3)	106 (18.3)
Ratio (%) profiles/isolates	24.7	29.0	34.8	36.4	40.1	55.8	28.0	35.3	28.3

*Numbers in bold correspond to values greater than 50% of the maximum possible ones.*

### MDR of Ampicillin-Resistant Isolates

**FIGURE 3:**
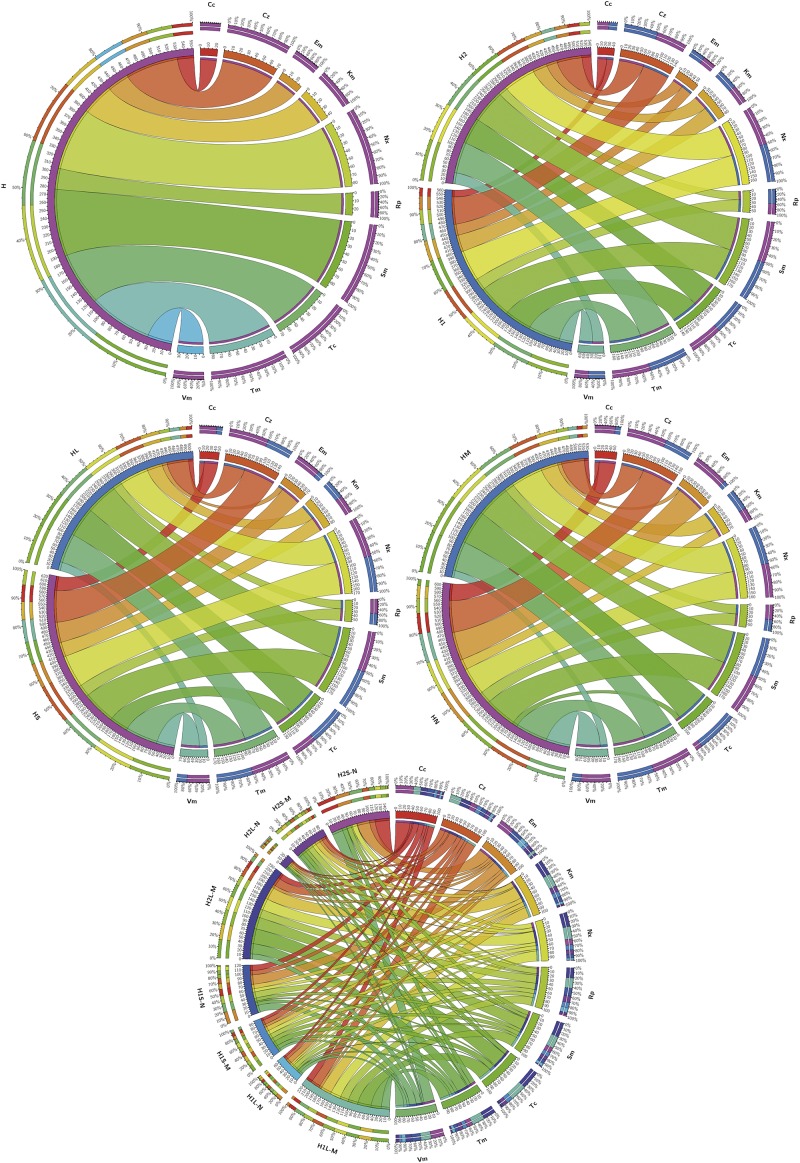
Prevalence of isolates resistant to the different antibiotics tested in the indicated groups of isolates. Considered groups of isolates were H: all, H1 and H2: all from both sampling sites independently, HL and HS: all from waters and sediments, respectively, HM and HN: all obtained from marine and nutritive media, respectively, and H1L-M, H1L-N, H1S-M, H1S-N, H2L-M, H2L-N, H2S-M, and H2S-N: groups considering independently sampling zones, phases and media. Ribbons connect antibiotic to which resistant isolates have been found and the groups of isolates in which they were found. Ribbons wideness represents percentages of bacteria resistant to the corresponding antibiotic among the isolates of the corresponding considered group.

Figure 3 summarizes the prevalence of resistances in the different groups of isolates considered and Table 2 shows the order of prevalences. Distributions of resistants to each antibiotic were significantly different among the samples (H1L, H2L, H1S, and H2S) and with respect to the culture media for almost all antibiotics (*p* < 0.05).

**TABLE 2:** Order of prevalence of resistance to individual antibiotics in the indicated groups of isolates.

**Group of isolates**	**Order of prevalence**
H	Sm > Tm = Nx > Tc > Cz > Km > Vm > Em > Rp > Cc
HL	Sm > Tc > Tm > Nx > Cz > Km > Rp > Vm > Em > Cc
HS	Sm > Nx > Tm > Cz > Em > Tc > Vm > Km > Cc > Rp
H1	Sm > Tm > Nx > Cz > Tc > Km > Vm > Rp > Em > Cc
H2	Sm > Nx > Tm > Tc > Cz > Km > Vm > Em > Cc > Rp
H1L	Sm > Tm > Nx > Tc > Cz > Km > Vm > Rp > Em > Cc
H1S	Sm > Nx > Cz > Tm > Tc > Km > Em > Vm > Rp > Cc
H2L	Sm > Tc > Nx > Tm > Cz > Km > Rp > Vm > Cc > Em
H2S	Tm > Nx > Sm > Cz > Em > Vm > Cc > Tc > Km > Rp

A total of 143 different resistance profiles were identified (Supplementary Table S4), with 2.1% of resistants only to Ap, and 2.6% to all the antibiotics. The most frequent profiles included resistance to eight antibiotics (128 isolates and 22 profiles), while the most diverse included resistance to 6 or 7 antibiotics (26 profiles each). 92.7% of the isolates were MRB [resistance to 4 or more antibiotic classes (Narciso-da-Rocha and Manaia, 2016)]. Table 1 summarizes the profiles/isolates ratios in each group.

The calculated MAR index medians (*x̃*) for considered groups of isolates (Table 1) were 0.636 for the whole set of isolates, H1 and H2. There was no statistically significant difference between water samples (HL, H1L, H2L) (*x̃* = 0.545, *p* > 0.05); however, sediments showed significantly higher values than the corresponding water: HS (*x̃* = 0.727, *p* < 0.001), H1S (*x̃* = 0.636, *p* = 0.001), and H2S (*x̃* = 0.727, *p* < 0.001). Distributions of MAR values of isolates from water and sediments of both sampling sites (H1L, H1S, H2L, H2S) were significantly different (*p* < 0.001) as well as when considering culture media (H1-M, H1-N, H2-M, H2-N) (*p* < 0.001).

### Phylogenetic Identification of Bacterial Isolates

Partial 16S rRNA sequences of the 345 isolates considered different by their MDR profiles and colony morphology were obtained, but phylogenetic assignation of some of them sometimes gave different results, mostly at the species level, when comparisons were made using either sequences at GenBank or phylogenetic trees (Supplementary Figures S1–S7); however, most affiliations were coincident at the genus level (Supplementary Table S5).

**FIGURE 4:**
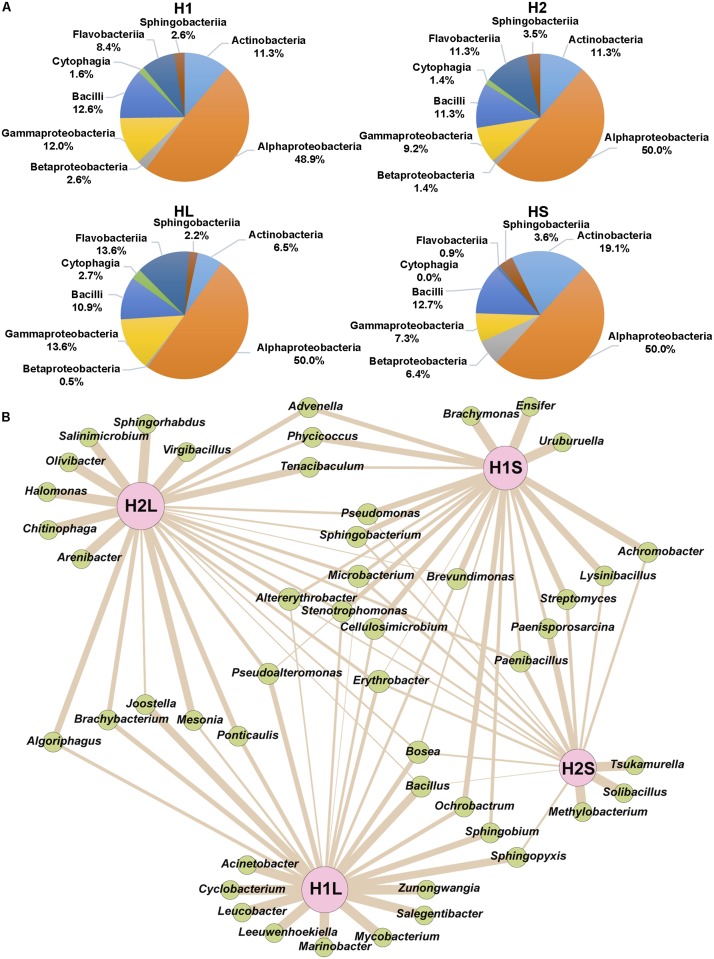
Distribution of phylogenetically identified isolates by class and genus in the indicated samples. **(A)** Classes, **(B)** Genera; wideness of each connector bar of the network is proportional to the number of isolates in each genus.

From H1 and H2 areas, 168 and 141 isolates, respectively, were considered to be different based on sample origin, multi-resistance profiles and 16S rDNA sequences. These isolates were assigned to phyla Proteobacteria, Bacteroidetes, Firmicutes, and Actinobacteria and classes Alphaproteobacteria, Bacilli, Gammaproteobacteria, Actinobacteria, Flavobacteriia, Betaproteobacteria, Sphingobacteriia, and Cytophagia (Figure 4A). Moreover, 48 different genera were identified, 52.1% were ubiquitous, 27.1% specifically of H1, and 20.8% of H2 (Figure 4B). Alphaproteobacteria showed the greatest diversity in genera and Cytophagia the least.

### Levels of MDR Among Phylogenetically Ascribed Isolates

Based on MAR values the level of MDR was: Actinobacteria = Bacteroidetes (*x̃* = 0.727) > Proteobacteria (*x̃* = 0.636) > Firmicutes (*x̃* = 0.455). At the Class level: Betaproteobacteria (*x̃* = 0.909) > Sphingobacteriia (*x̃* = 0.773) > Actinobacteria = Flavobacteriia (*x̃* = 0.727) > Alphaproteobacteria = Gammaproteobacteria (*x̃* = 0.636) > Bacilli = Cytophagia (*x̃* = 0.455). At the genus level, the highest was for *Stenotrophomonas* (*x̃* = 1.000) and the lowest was for *Pseudomonas* (*x̃* = 0.273). All *Ochrobactrum*, *Stenotrophomonas*, and *Streptomyces* were resistant to at least six antibiotics.

**FIGURE 5:**
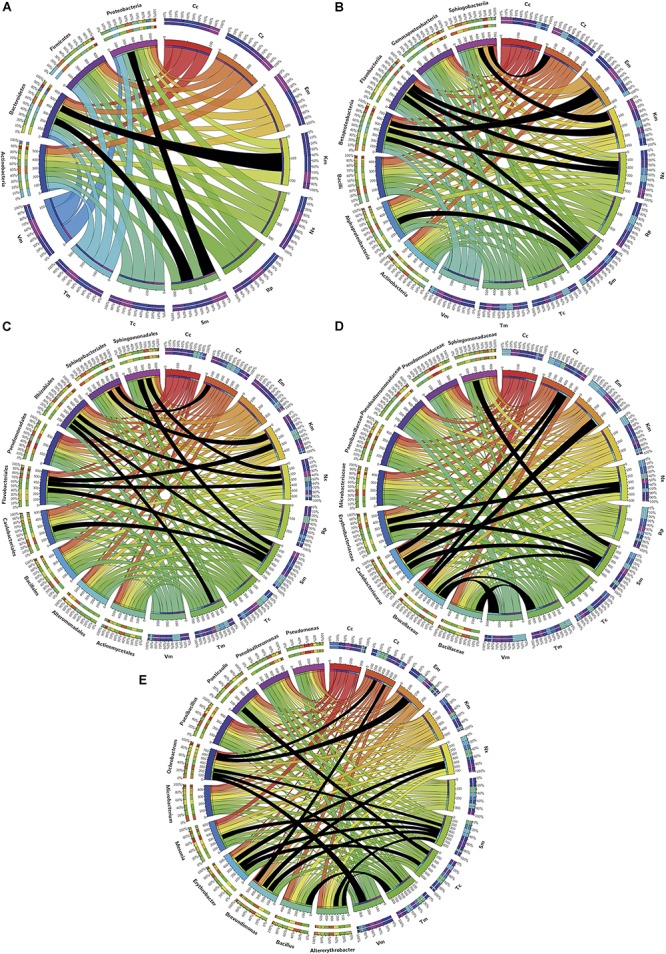
Distribution among phylogenetic groups of isolates resistant to the tested antibiotics. Graphs correspond to **(A)** phylum, **(B)** class, **(C)** order, **(D)** family, and **(E)** genus. Ribbons connect the antibiotic to which resistance was detected with the phylogenetic group to which resistant isolates were assigned. The wideness of each ribbon represents the percentage of isolates within each phylogenetic group resistant to the antibiotic that it connects to. Black ribbons correspond to percentages of isolates ≥90%.

Sm^R^ was the most frequently observed and Cc^R^ the least observed. The prevalent resistances (Figure 5) for the phylum/class Actinobacteria were Km^R^ and Tm^R^, and the less common were Em^R^ and Vm^R^. For Bacteroidetes, Km^R^ and Sm^R^ were the most frequent, while Em^R^ was the least. For Firmicutes, Tm^R^ and Vm^R^ were the most and least frequently found, respectively. For Proteobacteria, the most prevalent was Sm^R^ and the least was Cc^R^.

### Putative Intrinsic or Acquired Resistance of the Isolates

Resistance to a specific antibiotic was considered as putatively intrinsic in a particular phylogenetic group given the high majority (≥90%) of isolates that belong to that group were resistant (Figure 5). Thus, at the phylum level (Figure 5A), Bacteroidetes were intrinsically resistant to Km, Proteobacteria to Sm, and InR was neither observed in Firmicutes down to class nor in Actinobacteria down to genus.

At the class level (Figure 5B), Alphaproteobacteria showed InR only to Sm, and Betaproteobacteria to Em, Nx, and Sm, but for Gammaproteobacteria no InR was detected at this level or down to genus. Class/Order Flavobacteriia/Flavobacteriales showed InR to Km, Nx and Sm and class Sphingobacteriia to Cz and Km.

At the order level (Figure 5C), Caulobacteriales showed InR to Sm, Rhizobiales to Nx, Sm and Tm, and Sphingomonadales to Nx and Sm.

At the family level (Figure 5D), considering only those with more than one genus or species, Bacillaceae isolates appeared intrinsically resistant to Tm; Brucellaceae to Cz, Em, Sm, Tm, and Vm; Caulobacteriaceae to Cz, Nx and Sm; Erythrobacteriaceae to Nx and Sm; and Sphingomonadaceae to Sm.

Five genera of Alphaproteobacteria showed InR: *Altererythrobacter* to Sm and Tm; *Brevundimonas* to Cz, Nx, and Sm; *Erythrobacter* to Nx, Sm, and Tc; *Ochrobactrum* to Cz, Em, Sm, Tm, and Vm; *Ponticaulis* only to Sm; all *Bacillus* isolates were resistant to Tm; and all *Mesonia* isolates to Sm. No genus showed InR to Cc, Km, or Rp; and all isolates of the genera *Altererythrobacter*, *Bacillus*, and *Mesonia* were susceptible to Cc, Vm, and Em, respectively (Figure 5E).

### Correlations of Resistances Pairs in Each Phylogenetic Group

The kappa correlation index (κ) for pairs of antibiotic resistances was determined for the whole set of isolates and for the phylogenetic groups that included at least eight isolates.

**FIGURE 6:**
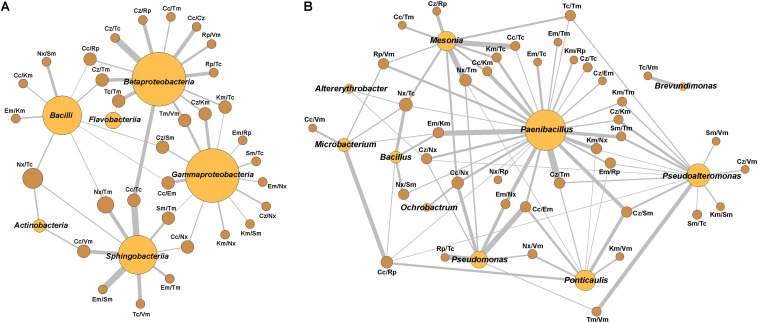
Network relationships of the best-correlated pairs of antibiotic resistances with the class or genus in which they were found. **(A)** Class, **(B)** Genus. The wideness of each connecting bar is proportional to the κ index value. Spheres size is proportional to the number of best-correlated (κ > 0.4) resistances pairs in each class or genus.

Considering all the isolates, no significant correlation appeared, but specific phylogenetic groups showed several positive and negative correlations. Only some of the positive ones reached κ > 0.400, corresponding to moderate and up to perfect correlations (κ = 0.810–1.000) (Supplementary Table S6). Perfect correlations appeared for Cz^R^/Tc^R^ in Betaproteobacteria and Cc^R^/Tc^R^ and Em^R^/Sm^R^ in Sphingobacteriia. At the genus level, these correlations were found in *Mesonia* for Cc^R^/Tc^R^ and Nx^R^/Tm^R^, in *Microbacterium* for Cc^R^/Rp^R^, in *Paenibacillus* for Cc^R^/Em^R^, Cc^R^/Km^R^, Em^R^/Nx^R^, Km^R^/Nx^R^, Cz^R^/Tm^R^ and Em^R^/Km^R^, in *Pseudoalteromonas* for Tm^R^/Vm^R^, and, finally, in *Pseudomonas* for Cc^R^/Em^R^ and Rp^R^/Tc^R^. These correlations configure networks, which were represented for κ values higher than 0.4 (Figure 6). In these networks, some pairs of resistances with moderate or optimal correlations are shared by several genera while others appeared only in specific ones.

## Discussion

### Environmental Conditions of the Sampling Zones

The samples used in this work contained heavy metals with a slightly higher level of the most abundant elements in the H1 area and with lower salinity and conductivity when compared to the H2 area. This result may be due to the effect of a higher influence from the Tinto River water, which constitutes the leading heavy metal source of the H1 station. The industrial plants around H2 also dump metals and metalloids into the water but with a lower contribution than from the rivers (Pérez-López et al., 2011). The concentration of metals in the estuarine water is much lower than what is usually found in the Tinto River, which is probably the result of the dilution effect from seawater entry. Also, the higher pH of the water could trigger the precipitation of some metals (Rodgers et al., 2019). The higher concentration of phosphor in H1S versus H2S may be due to the input of phosphogypsum-rich wastewater from a nearby fertilizer plant (Sainz et al., 2004), which recently was shown to contaminate the estuary with heavy metals through a weathering process (Papaslioti et al., 2018). The presence of other contaminants in the estuary was not the aim of this study; however, it is advisable, based on the discharge of effluents from the Huelva WWTP into the estuary, which can provide contamination by antibiotics and other pharmaceuticals as has been detected in the effluents from urban WWTPs around the world. Considering the differences observed in metals composition and the physicochemical characteristics of both sampling zones, a differential impact of these factors on the biodiversity could not be ruled out.

### Abundance of Culturable Bacteria

As customarily expected by the marine origin of most waters arriving into the estuary, the abundance of culturable bacteria was higher on the marine medium than on the nutritive medium. Also, in both phases, the abundance of bacteria was higher in H1 than in H2. Our results are also in line with previous reports that have shown a higher bacterial richness in sediments than in the water column of several environments (Ye et al., 2009; Dai et al., 2016; Narciso-da-Rocha and Manaia, 2016). The lower abundances found in the H2 samples on both media might be due to the possible presence of toxic pollutants emitted by nearby industries. Additionally, H2 is closer to the WWTP, which could discharge antibiotics into the estuary that may not have been completely removed during the sewage treatment as reported in other cases (Zhou et al., 2013; Tran et al., 2016). The difference in abundance between the phases indicates the accumulation of bacteria in the sediments with higher stability in the estuarine system. The sediments offer protection against biotic and abiotic stresses like tidal water movements. Bacteria counts of sediments may even be underestimated as some studies claim (Hassard et al., 2017).

### Antibiotic Resistance Levels and Diversity in the Estuary

Eleven antibiotics representatives of different mechanisms of action were chosen. The antibiotic concentrations to use for susceptibility testing of environmental bacteria have not been standardized; and the reported studies used different concentrations (D’Costa et al., 2006; Bhullar et al., 2012; Voolaid et al., 2012; Narciso-da-Rocha and Manaia, 2016; Jardine et al., 2017). In this study, we wanted to focus on the ARs that might be relevant for the ecosystem and for clinical microbiology. So, concentrations related to the reported resistance/susceptibility breakpoints for various clinical bacteria according to EUCAST (European Committee on Antimicrobial Susceptibility Testing [EUCAST], 2013) were used. However, the used values were slightly higher than the breakpoints of EUCAST to avoid overestimations of the resistance level.

Resistants to Km, Nx, Sm, Tc, or Tm were more abundant among bacteria growing on the marine medium than on the nutritive medium for all samples. Furthermore, Cc^R^, Em^R^, or Rp^R^ were rarely found in bacteria growing on marine media. These results indicated that the population of bacteria growing on one or the other media are not the same and carry determinants for different ARs. The highest difference between media was for Tc^R^, common in isolates from the marine medium. Several studies have shown a great abundance of Tc^R^ genes in different environments including marine sediments (Yang J. et al., 2013), river estuary (Lin et al., 2015), or wastewater and activated sludge (Auerbach et al., 2007; Zhang and Zhang, 2011; Yang Y. et al., 2013). Moreover, Tc has been reported to be non-biodegradable in activated sludge or different conditions (Prado et al., 2009; Cetecioglu et al., 2014), so it needs special treatment for degradation in water (Wang et al., 2011) and sediments (Chang and Ren, 2015); these factors could help with the selection of Tc^R^ bacteria.

### Prevalence of Resistance to Individual Antibiotics Among Ap^R^ Bacterial Isolates

Resistance to Sm was the most prevalent considering all isolates (H), those from liquid (HL) or sediment (HS) phases or H1 and H2 separately. Tc^R^ was prevalent in most of the groups of isolates obtained on the marine medium except isolates from H2S-M and HS-M, which had a slightly higher prevalence of Nx^R^. Likewise, Tm^R^ was prevalent on those on the nutritive medium except for the isolates from H1S-N and HS-N for which Cz^R^ was more prevalent. These data show that, in general, Sm^R^, Tc^R^, and Tm^R^ were the prevalent resistances among the isolates mostly in agreement with data from the direct plating of original samples. Thus, the isolates obtained are a good representation of the estuarine culturable ARB. The quantitative differential presence of each bacterium in the original samples was partially deconvoluted when colonies were selected in the isolation step and this might account for the differences observed.

Some studies have shown that streptomycin can persist in aquatic environments for some time and its degradation products still maintain certain antibacterial activity (Shen et al., 2017). On the other hand, selection of Sm^R^ bacteria in *in vitro* evolution experiments with low concentrations of antibiotic has been observed even after only 96 h (Spagnolo et al., 2015). Moreover, some studies have shown a relationship between heavy metal and Sm resistances (Matyar et al., 2014) or aminoglycoside resistance genes (Zhao et al., 2019) and plasmids have been found to contain determinants for co-resistance to Sm and Cu (Sundin and Bender, 1996). So, one possible reason for the high prevalence of Sm^R^ in the estuary’s isolates could be the co-selection due to the presence of Cu or other heavy metals. Also, correlations of Tc^R^ genes prevalences with the presence of heavy metals, also present in the Huelva estuary, such as Cu, Mn, Ni, and Zn have been reported (Knapp et al., 2017). Recently, in a paleontological study of sediments contaminated with Zn, bacteria were found to share Tm^R^ with Zn resistance, which was related to Zn concentration (Dickinson et al., 2019). The co-selection for Zn^R^ likely might be one of the causes for the prevalence of Tm^R^ in bacteria from the Huelva estuary. In addition to possible effects by other contaminants, different studies have shown the coexistence, and sometimes a correlation, of AR and heavy metals resistances in waters and sediments [reviewed by Nguyen et al. (2019)].

Further molecular studies on the genetics determinants for Sm^R^, Tc^R^, and Tm^R^ in the isolates will give insights on the causes of such high prevalence and mechanisms of co-selection.

### MDR Levels and Profiles Diversity Among Ap^R^ Bacterial Isolates

Multi-drug resistance levels were calculated for different groups of isolates using the MAR index median as the comparative parameter. The diversity of multi-resistance profiles were assessed as the ratio number-of-profiles/number-of-isolates. These parameters could give insights on the availability of resistance’s determinants, some of which might be transmissible, as well as on the diversity of putative groups of them that might be transmitted together.

Based on the diversity of colony morphologies and sample origin, 579 Ap^R^-bacteria were isolated to determine the level and profiles of MDR. This is one of the highest numbers of isolates analyzed in similar studies using culturable bacteria. The test of resistance to the other 10 antibiotics carried out on this collection showed diverse resistance profiles. 92.7% of the whole set of isolates were resistant to 4 or more classes of antibiotics and the median of MAR indexes corresponded to resistance to 7/11 of the tested antibiotics. These results represent outstanding levels of multi-resistance and as far as we know, one of the highest measured up to now in a non-clinical setting.

Higher MDR levels were found in isolates from sediments than from those in waters when considering all isolates from the estuary (H), or each individual zone (H1 or H2). This difference between phases could be due to the lower mobility of sediments and the possible accumulation of antibiotics, metals, or other pollutants (including ARGs) that may facilitate the selection of resistant bacterial populations, as has been proposed for other systems (Rodgers et al., 2019). Furthermore, the accumulation of resistant bacteria in sediments could facilitate bacterial cell contact; and, therefore, to increase the efficiency of HGT (Zhang et al., 2018).

The diversity of MDR profiles is higher in sediments than in waters when considering all isolates (H) or those from H1 zone alone. When isolates were segregated by the culture medium used for isolation, only those on the marine medium showed higher profile diversity in sediments than in waters.

When comparing whole sets of isolates from the two sampling zones (H1 and H2), no difference was found in the MDR level. However, when isolates were segregated by the culture medium a significant difference appeared between the isolates on the nutritive medium, with the highest value for those of H2.

Thus, in general, the isolates on the nutritive medium from sediments were resistant to more antibiotics and showed less diverse profiles than those from water samples. However, those obtained on the marine medium showed higher levels of multi-resistance and also higher profiles diversity when considering the isolates obtained from sediments versus those isolated from water samples.

To try to explain the observed differences between the levels and diversity of MDR for the isolates from sediments of H1 and H2 zones on the nutritive medium, our primary hypothesis was the more direct influence of the effluents of the WWTP on H2. This might be responsible for the higher MDR in the isolates on the nutritive medium since the effects of WWTP effluents on bacterial resistance have been shown in several studies (Guo et al., 2017; Manaia et al., 2018). Nevertheless, we noticed that a number of isolates from the sample H2S-N had the same MDR profile and had similar colony morphology. They were initially selected because they appeared in high numbers in the original selection step. When only one of these isolates was considered, the MAR median for the H2S-N sample decreased to 0.636, with no difference with H1S-N. The ratio profiles/isolates became in this way 64.7 and 46.2% for H2S-N and H2S, respectively; and were more similar to the behavior of H1 isolates. Hence, the overrepresentation of a particular bacterium with a high MAR value was biasing the MAR value of the corresponding sample. This bias stresses the need for deconvolution of the isolates to detect the real levels of resistance and diversity of MDR. We thus concluded that not much difference existed between the MDR levels of isolates on the nutritive medium from the sediments of both sampling zones.

### Biodiversity of Culturable MRB From the Estuary

16S rDNA sequences from 345 isolates were firstly compared with those available in GenBank database and assigned to eight phylogenetic classes found in both sampling areas in similar proportions. Alphaproteobacteria constituted around 50% of the isolates in each zone. This percentage suggests that the tidal movement of water in the estuary could play an essential role in the homogenization of the bacterial composition at both sampling sites. However, analysis at the genus level showed higher diversity differences and stressed the need to phylogenetically ascribe down to the lowest possible phylogenetic level to obtain a more accurate picture of diversity distribution differences. A higher difference in the classes’ distribution was observed between liquid and sediment phases: Actinobacteria and Betaproteobacteria were more abundant in the sediments; and Gammaproteobacteria and Flavobacteriia in waters.

A more detailed phylogenetic analysis using trees built with sequences at RDP identified 48 genera. Although most of the genera were common to both sampling zones, some were found only in one zone, which perhaps reflects physicochemical differences between them.

The phylogenetic composition of the culturable Ap^R^ isolates found in this study is similar to the diversity of bacterioplankton detected in other estuaries or marine environments when compared at the phylum and class levels (Selje et al., 2005; Jamieson et al., 2012; Du et al., 2013; Sjöstedt et al., 2014; Vaz-Moreira et al., 2014). However, a deeper level of phylogenetic assignation is needed in most studies to be able to make further comparisons.

The majority of the identified species have not been described as pathogens. However, some of them may be considered as opportunistic pathogens or have been found in some sporadic cases of infections (Supplementary Table S4). So, some health concerns should be considered for the bacteria in the estuary due to their high level of multi-resistance. Since the phylogenetic diversity of MDR bacteria found in the estuary is high, the possible dissemination of AR or MDR to diverse other bacteria is also likely.

### Intrinsic and Acquired Resistance

Intrinsic resistance is the most common form of resistance in some bacteria (Cox and Wright, 2013). In this study, the putative InR of four phyla, seven classes, and 11 genera represented by eight or more isolates were analyzed and putative InR was found in Bacteroidetes and Proteobacteria. Different results have been found in other studies (Walsh and Duffy, 2013). Hence, a selection of bacteria that depends on the presence of different substances and physicochemical conditions should have occurred in the Huelva estuary.

At the class level, Gammaproteobacteria did not show any InR, while Alphaproteobacteria, Betaproteobacteria, and Flavobacteriia showed InR to Sm. These last two also shared InR to Nx. On the other hand, Flavobacteriia and Sphingobacteriia shared InR to Km. Moreover InR to Em was only found in Betaproteobacteria and Cz in Sphingobacteriia.

Among the genera that did not show any InR, the results for *Pseudomonas* contrast with the case of clinical *P. aeruginosa*, which is intrinsically resistant to several antibiotics (Alvarez-Ortega et al., 2011; Breidenstein et al., 2011; Vaz-Moreira et al., 2012). Prevalence of antibiotic resistance in environmental *Pseudomonas* is related to the phylogenetic species level rather than to the genus level (Vaz-Moreira et al., 2012), and for environmental *P. aeruginosa* is lower than for clinical isolates (Liew et al., 2019). In a recent study (Luczkiewicz et al., 2015), *Pseudomonas* spp. were isolated from influents, effluents, and receiving marine-water from a WWTP, recovering fewer isolates from marine water than from the water from the WWTP. Furthermore, *P. putida* was the predominant species in all samples whereas *P. aeruginosa* was the second major species in the WWTP effluents (enriched from the influents water), but the latter was found in a much lower proportion in receiving water. This effect could explain the lack of this species among our isolates. Additionally, in the above-referred report, *P. putida* and *P. aeruginosa* isolates were tested for AR to 14 antibiotics, but no InR would have been found under the criterion used by us. Our isolates of the remaining analyzed genera showed putative InR to one; as *Bacillus*, *Mesonia*, and *Ponticaulis;* or several antibiotics. The genus with the highest numbers was *Ochrobactrum* that had five InRs.

None of the phylogenetic groups showed InR to Cc or Rp, and the number of isolates resistant to these antibiotics was generally low. The percentage of Cc^R^-isolates reached up to 50% only for *Pseudomonas*. These low levels of resistants may be a consequence of the limited use of Cc because of its toxicity (Ingebrigtsen et al., 2017); and, therefore, the existence of low selective pressure for this antibiotic in the environment. It has been shown that Rp^R^ in pathogenic bacteria has a high metabolic cost, which significantly affects the fitness of the bacteria making their ARGs uncommon (Hall et al., 2011). The fitness cost might have conditioned most isolates, but more than 80% of the *Ochrobactrum* isolates showed Rp^R^ and seemed to be able to work around these fitness costs.

Non-intrinsic resistances should be considered as acquired independently of the mechanism by which they were obtained (mutation or HGT).

### Correlation of the Presence of Pairs of Antibiotic Acquired Resistances in Phylogenetic Groups of Isolates

In not considering the putative InRs discussed above, the correlations of resistances to pairs of antibiotics were low when all available isolates were considered. These data indicated a low probability of cross or co-resistance to any of the tested pairs of antibiotics in the whole bacterial population. However, some moderate to almost perfect correlations were found at the class level, more frequently in Beta or Gammaproteobacteria, and strong ones at the genus level.

Strong positive correlations were observed at the genus level, particularly in *Paenibacillus.* In the network built based on κ>0.4 values, *Paenibacillus* appears central sharing many correlated pairs of resistances with other genera suggesting possible sharing of MGEs. Some of these elements have been described in this genus (Pednekar et al., 2010; Soundarapandian et al., 2013). The presence of these elements indicates that this genus might act as an ARG exchanger among different genera, and perhaps, plays a crucial role in the resistance dissemination in the Huelva estuary. Apart from this, an accumulation of ARGs in this genus has been described but classified as intrinsic (Pawlowski et al., 2016). Our hypothesis about *Paenibacillus*’ role in the ecosystem deserves to be studied in the future because specific published data about this genus suggest a great versatility among its members. First, this genus contains a high number of species and new ones are described frequently (Sáez-Niero et al., 2017), so this genus displays a high intra-genus genetic variability. In the second place, *Paenibacillus* species have been described in a variety of environments, some of them interacting with other organisms (Finkelshtein et al., 2015; Grady et al., 2016; Xie et al., 2016), which suggests their potential for affecting ecology. Also, strains of the same species showed numerous variations in genome sequences with a high rate of recombination, which makes them prone to exchange DNA fragments by horizontal genetic exchange (Xie et al., 2016; Xu et al., 2017). Likewise, several species of the genus produce diverse antibiotic substances (Zhao et al., 2018; Pasari et al., 2019), which could make them players in the shaping of their ecosystems. Furthermore, *Paenibacillus* genomes have a plethora of genes for adaptation, some of which considered to be acquired by HGT (Xie et al., 2016; Xu et al., 2017). Moreover, some studies have considered that the species of the genus evolve not as individual species, but inter-related in a pan-genomic evolution model (Xu et al., 2017). Finally, the resistance elements found in genomes of some *Paenibacillu*s species could be the origin of resistance elements found in other genera (Guardabassi et al., 2005; Liu et al., 2016); and, thus, they show mobilization potential. All this suggests that this genus may play a crucial role in any biological system because of its capabilities to adapt and interact with other organisms. Considering that some *Paenibacillus* species can infect humans, animals, and plants and are quite ubiquitous, they could be an essential and currently unconsidered threat when present in an ecosystem, either directly or by its putative ARGs exchanger role, which could affect the ecology of the systems where they are present. A recent study by Wright’s group (Pawlowski et al., 2018) also suggested a role for Paenibacillaceae bacteria in environmental mobilization and dissemination of resistance genes.

## Conclusion

In conclusion, the culturable bacterial population from the Huelva estuary contains highly-multi-resistant bacteria some of which have been isolated and characterized for MDR profiles to 11 antibiotics, which show a diversity of combinations of resistances. The analysis of AR of the phylogenetically ascribed isolates has shown the existence of putative InR in most of the genera. However, some other ARs may be acquired and some are perhaps transferrable. Moderated to optimal correlations of pairs of resistances in certain phylogenetic groups suggests that these resistances could be transferred among their members. In particular, *Paenibacillus* showed a large number of these correlations, many of which are shared by other genera as a network study has shown. From these results, a hypothesis for future studies can be outlined in which *Paenibacillus* could have a role as a player in the dissemination of resistance in this environment.

## Data Availability Statement

The 16S rRNA sequences generated for this study can be found in the GenBank (accession numbers LT601033-LT601378).

## Author Contributions

JA contributed to the conception and design of the study, sampling, and wrote the manuscript. BE-C, HM-F, and JA performed the experiments and analyzed the data. BE-C and JA performed the statistical analysis. All authors contributed to the manuscript revision, read and approved the submitted version.

## Conflict of Interest

The authors declare that the research was conducted in the absence of any commercial or financial relationships that could be construed as a potential conflict of interest.

## Footnotes

1 https://www.ecdc.europa.eu/en/antimicrobial-consumption/database/country-overview

2 https://www.iagua.es/data/infraestructuras/edar/huelva

3 http://www.uam.es/UAM/SIdI/1242663052047.htm?language=es

4 http://www.clustal.org/clustal2/

5 http://circos.ca/

6 https://gephi.org/

## References

[B1] Altschul S. F. Gish W. Miller W. Myers E. W. Lipman D. J. (1990). Basic local alignment search tool. *J. Mol. Biol.* 215 403–410. 10.1016/S0022-2836(05)80360-80362 2231712

[B2] Alvarez-Ortega C. Wiegand I. Olivares J. Hancock R. E. Martínez J. L. (2011). The intrinsic resistome of *Pseudomonas aeruginosa* to β-lactams. *Virulence* 2 144–146. 10.4161/viru.2.2.15014 21304266

[B3] Amils R. (2016). Lessons learned from thirty years of geomicrobiological studies of Río Tinto. *Res. Microbiol.* 167 539–545. 10.1016/j.resmic.2016.06.001 27349346

[B4] Andersson D. I. Hughes D. (2014). Microbiological effects of sublethal levels of antibiotics. *Nat. Rev. Microbiol.* 12 465–478. 10.1038/nrmicro3270 24861036

[B5] Auerbach E. A. Seyfried E. E. McMahon K. D. (2007). Tetracycline resistance genes in activated sludge wastewater treatment plants. *Water Res.* 41 1143–1151. 10.1016/j.watres.2006.11.045 17239919

[B6] Baharoglu Z. Mazel D. (2011). *Vibrio cholerae* triggers SOS and mutagenesis in response to a wide range of antibiotics: a route towards multiresistance. *Antimicrob. Agents Chemother.* 55 2438–2441. 10.1128/AAC.01549-1510 21300836 PMC3088271

[B7] Baker-Austin C. Wright M. S. Stepanauskas R. McArthur J. V. (2006). Co-selection of antibiotic and metal resistance. *Trends Microbiol.* 14 176–182. 10.1016/j.tim.2006.02.006 16537105

[B8] Barancheshme F. Munir M. (2018). Strategies to combat antibiotic resistance in the waste- water plants. *Front. Microbiol.* 8:2603 10.3389/fmicb.2017.02603 PMC5776126 29387043

[B9] Bhullar K. Waglechner N. Pawlowski A. Koteva K. Banks E. D. Johnston M. D. (2012). Antibiotic resistance is prevalent in an isolated cave microbiome. *PLoS One* 7:e34953. 10.1371/journal.pone.0034953 22509370 PMC3324550

[B10] Boxall A. Wilkinson J. (2019). “Identifying hotspots of resistance selection from antibiotic exposure in urban environments around the World. Communication 130,” in *SETAC Europe 29th Annual Meeting* (Helsinki).

[B11] Breidenstein E. B. de la Fuente-Núñez C. Hancock R. E. (2011). *Pseudomonas aeruginosa*: all roads lead to resistance. *Trends Microbiol.* 19 419–426. 10.1016/j.tim.2011.04.005 21664819

[B12] Brown-Jaque M. Calero-Cáceres W. Muniesa M. (2015). Transfer of antibiotic-resistance genes via phage-related mobile elements. *Plasmid* 79 1–7. 10.1016/j.plasmid.2015.01.001 25597519

[B13] Cambray G. Guerout A. M. Mazel D. (2010). Integrons. *Annu. Rev. Genet.* 44 141–166. 10.1146/annurev-genet-102209-163504 20707672

[B14] Carraro N. Rivard N. Burrus V. Ceccarelli D. (2017). Mobilizable genomic islands, different strategies for the dissemination of multidrug resistance and other adaptive traits. *Mob. Genet. Elements* 7 1–6. 10.1080/2159256X.2017.1304193 28439449 PMC5397120

[B15] Carraro N. Rivard N. Ceccarelli D. Colwell R. R. Burrus V. (2016). IncA/C conjugative plasmids mobilize a new family of multidrug resistance islands in clinical *Vibrio cholerae* non-O1/non-O139 isolates from Haiti. *MBio* 7 e509–e516. 10.1128/mBio.00509-516 27435459 PMC4958241

[B16] Cetecioglu Z. Ince B. Azman S. Ince O. (2014). Biodegradation of tetracycline under various conditions and effects on microbial community. *Appl. Biochem. Biotech.* 172 631–640. 10.1007/s12010-013-0559-556 24104689

[B17] Chamosa L. S. Aìlvarez V. E. Nardelli M. Quiroga M. P. Cassini M. H. Centroìn D. (2017). Lateral antimicrobial resistance genetic transfer is active in the open environment. *Sci. Rep* 7:513. 10.1038/s41598-017-00600-602 28364120 PMC5428826

[B18] Chang B.-V. Ren Y.-L. (2015). Biodegradation of three tetracyclines in river sediment. *Ecol.Eng.* 75 272–277. 10.1016/j.ecoleng.2014.11.039

[B19] Chen B. He R. Yuan K. Chen E. Lin L. Chen X. (2017). Polycyclic aromatic hydrocarbons (PAHs) enriching antibiotic resistance genes (ARGs) in the soils. *Environ. Pollut.* 220 (Pt B), 1005–1013. 10.1016/j.envpol.2016.11.047 27876418

[B20] Cole J. R. Wang Q. Fish J. A. Chai B. McGarrell D. M. Sun Y. (2014). Ribosomal database project: data and tools for high throughput rRNA analysis. *Nucl. Acids Res.* 42 D633–D642. 10.1093/nar/gkt1244 24288368 PMC3965039

[B21] Cox G. Wright G. D. (2013). Intrinsic antibiotic resistance: mechanisms, origins, challenges and solutions. *Int. J. Med. Microbiol.* 303 287–292. 10.1016/j.ijmm.2013.02.009 23499305

[B22] Currie C. R. Scott J. A. Summerbell R. C. Malloch D. (1999). Fungus-growing ants use antibiotic-producing bacteria to control garden parasites. *Nature* 398 701–704. 10.1038/19519 19270078

[B23] Cycon M. Mrozik A. Piotrowska-Seget Z. (2019). Antibiotics in the soil environment,-degradation and their impact on microbial activity and diversity. *Front. Microbiol.* 10:338 10.3389/fmicb.2019.00338 PMC6418018 30906284

[B24] Dai Y. Yang Y. Wu Z. Feng Q. Xie S. Liu Y. (2016). Spatiotemporal variation of planktonic and sediment bacterial assemblages in two plateau freshwater lakes at different trophic status. *Appl. Microbiol. Biotechnol.* 100 4161–4175. 10.1007/s00253-015-7253-7252 26711281

[B25] Danner M.-C. Robertson A. Behrends V. Reiss J. (2019). Antibiotic pollution in surface fresh waters: occurrence and effects. *Sci. Total Environ.* 664 793–804. 10.1016/j.scitotenv.2019.01.406 30763859

[B26] Davies J. Spiegelman G. B. Yim G. (2006). The world of subinhibitory antibiotic concentrations. *Curr. Opin. Microbiol.* 9 445–453. 10.1016/j.mib.2006.08.006 16942902

[B27] Davis R. A. Jr. Welty A. T. Borrego J. Morales J. A. Pendon J. G. Ryan J. G. (2000). Rio Tinto estuary (Spain): 5000 years of pollution. *Environ. Geol.* 39 1107–1116. 10.1007/s002549900096

[B28] D’Costa V. M. McGrann K. M. Hughes D. W. Wright G. D. (2006). Sampling the antibiotic resistome. *Science* 311 374–377. 10.1126/science.1120800 16424339

[B29] Dealtry S. Holmsgaard P. N. Dunon V. Jechalke S. Ding G. C. Krögerrecklenfort E. (2014). Shifts in abundance and diversity of mobile genetic elements after the introduction of diverse pesticides into an on-farm biopurification system over the course of a year. *Appl. Environ. Microbiol.* 80 4012–4020. 10.1128/AEM.04016-4013 24771027 PMC4054223

[B30] Devault A. M. Mortimer T. D. Kitchen A. Kiesewetter H. Enk J. M. Golding G. B. (2017). A molecular portrait of maternal sepsis from Byzantine Troy. *Elife* 6:e20983. 10.7554/eLife.20983 28072390 PMC5224923

[B31] Dickinson A. W. Power A. Hansen M. G. Brandt K. K. Piliposian G. Appleby P. (2019). Heavy metal pollution and co-selection for antibiotic resistance: a microbial palaeontology approach. *Environ. Int.* 132:105117. 10.1016/j.envint.2019.105117 31473413

[B32] Ding C. He J. (2010). Effect of antibiotics in the environment on microbial populations. *Appl. Microbiol. Biotechnol.* 87 925–941. 10.1007/s00253-010-2649-2645 20508933

[B33] Du J. Xiao K. Li L. Ding X. Liu H. Lu Y. (2013). Temporal and spatial diversity of bacterial communities in coastal waters of the South China Sea. *PLoS One* 8:e66968. 10.1371/journal.pone.0066968 23785512 PMC3681761

[B34] Eduardo-Correia B. (2016). *Abundancia, diversidad y perfiles de multirresitencia de bacterias cultivables resistentes a antibióticos en la Ría de Huelva y la Chorrera de Despeñalagua (Guadalajara).* Madrid: Universidad Autónoma de Madrid.

[B35] Elbaz-Poulichet F. Morley N. H. Cruzado A. Velasquez Z. Achterberg E. P. Braungardt C. B. (1999). Trace metal and nutrient distribution in an extremely low pH (2.5) river-estuarine system, the Ria of Huelva (South-West Spain). *Sci. Total Environ.* 227 73–83. 10.1016/S0048-9697(99)00006-6

[B36] Ellington M. J. Ekelund O. Aarestrup F. M. Canton R. Doumith M. Giske C. (2017). The role of whole genome sequencing in antimicrobial susceptibility testing of bacteria: report from the EUCAST Subcommittee. *Clin. Microbiol. Infect.* 23 2–22. 10.1016/j.cmi.2016.11.012 27890457

[B37] European Centre for Disease Prevention and Control [ECDC] (2017). *Summary of the Latest Data on Antibiotic Consumption in the European Union. ESAC-Net Surveillance Data.* Available at: https://www.ecdc.europa.eu/sites/default/files/documents/Final_2017_EAAD_ESAC-Net_Summary-edited%20-%20FINALwith%20erratum.pdf (accessed December 24, 2019).

[B38] European Committee on Antimicrobial Susceptibility Testing [EUCAST] (2013). *EUCAST Guidelines for Detection of Resistance Mechanisms and Specific Resistances of Clinical and/or Epidemiological Importance. Version 1.0.* Available at: http://www.eucast.org/fileadmin/src/media/PDFs/EUCAST_files/Resistance_mechanisms/EUCAST_detection_of_resistance_mechanisms_v1.0_20131211.pdf (accessed May 28, 2019).

[B39] Fajardo A. Linares J. F. Martínez J. L. (2009). Towards an ecological approach to antibiotics and antibiotic resistance genes. *Clin. Microbiol. Infect.* 1 14–16. 10.1111/j.1469-0691.2008.02688.x 19220346

[B40] Fajardo A. Martinez J. L. (2008). Antibiotics as signals that trigger specific bacterial responses. *Curr. Opin. Microbiol.* 11 161–167. 10.1016/j.mib.2008.02.006 18373943

[B41] Finkelshtein A. Roth D. Ben-Jacob E. Ingham C. J. (2015). Bacterial swarms recruit cargo bacteria to pave the way in toxic environments. *MBio* 6:e00074-15. 10.1128/mBio.00074-15 25968641 PMC4436059

[B42] Goneau L. W. Hannan T. J. MacPhee R. A. Schwartz D. J. Macklaim J. M. Gloor G. B. (2015). Subinhibitory antibiotic therapy alters recurrent urinary tract infection pathogenesis through modulation of bacterial virulence and host immunity. *MBio* 6:e00356-15. 10.1128/mBio.00356-315 25827417 PMC4453531

[B43] Grady E. N. MacDonald J. Liu L. Richman A. Yuan Z.-C. (2016). Current knowledge and perspectives of *Paenibacillus*: a review. *Microb. Cell Fact.* 15:203. 10.1186/s12934-016-0603-607 27905924 PMC5134293

[B44] Grenni P. Ancona V. Barra Caracciolo A. (2018). Ecological effects of antibiotics on natural ecosystems: a review. *Microchem. J.* 136 25–39. 10.1016/j.microc.2017.02.006

[B45] Guardabassi L. Perichon B. Van Heijenoort J. Blanot D. Courvalin P. (2005). Glycopeptide resistance *vanA* operons in *Paenibacillus* strains isolated from soil. *Antimicrob. Agents Chemother.* 49 4227–4233. 10.1128/AAC.49.10.4227-4233.2005 16189102 PMC1251550

[B46] Guo J. Li J. Chen H. Bond P. L. Yuan Z. (2017). Metagenomic analysis reveals wastewater treatment plants as hotspots of antibiotic resistance genes and mobile genetic elements. *Water Res.* 123 e468–e478. 10.1016/j.watres.2017.07.002 28689130

[B47] Gupta R. S. (2011). Origin of diderm (Gram-negative) bacteria: antibiotic selection pressure rather than endosymbiosis likely led to the evolution of bacterial cells with two membranes. *Antonie Van Leeuwenhoek* 100 171–182. 10.1007/s10482-011-9616-9618 21717204 PMC3133647

[B48] Gutierrez A. Laureti L. Crussard S. Abida H. Rodríguez-Rojas A. Blázquez J. (2013). β-lactam antibiotics promote bacterial mutagenesis via an RpoS-mediated reduction in replication fidelity. *Nat. Commun.* 4:1610. 10.1038/ncomms2607 23511474 PMC3615471

[B49] Haaber J. Penades J. R. Ingmer H. (2017). Transfer of antibiotic resistance in *Staphylococcus aureus*. *Trends Microbiol.* 25 893–905. 10.1016/j.tim.2017.05.011 28641931

[B50] Haas D. Défago G. (2005). Biological control of soil-borne pathogens by fluorescent pseudomonads. *Nat. Rev. Microbiol.* 3 307–319. 10.1038/nrmicro1129 15759041

[B51] Hall A. R. Iles J. C. MacLean R. C. (2011). The fitness cost of rifampicin resistance in *Pseudomonas aeruginosa* depends on demand for RNA Polymerase. *Genetics* 187 817–822. 10.1534/genetics.110.124628 21220359 PMC3063675

[B52] Hassard F. Andrews A. Jones D. L. Parsons L. Jones V. Cox B. A. (2017). Physicochemical factors influence the abundance and culturability of human enteric pathogens and fecal indicator organisms in estuarine water and sediment. *Front. Microbiol.* 8:1996. 10.3389/fmicb.2017.01996 29089931 PMC5650961

[B53] Hiltunen T. Virta M. Laine A.-L. (2017). Antibiotic resistance in the wild: an eco-evolutionary perspective. *Phil. Trans. R. Soc. B* 372:20160039. 10.1098/rstb.2016.0039 27920384 PMC5182435

[B54] Ingebrigtsen S. G. Didriksen A. Johannessen M. Škalko-Basnet N. Holsæter A. M. (2017). Old drug, new wrapping - A possible comeback for chloramphenicol? *Int. J. Pharm.* 526 538–546. 10.1016/j.ijpharm.2017.05.025 28506801

[B55] Jamieson R. E. Rogers A. D. Billett D. S. Smale D. A. Pearce D. A. (2012). Patterns of marine bacterioplankton biodiversity in the surface waters of the Scotia Arc, Southern Ocean. *FEMS Microbiol. Ecol.* 80 452–468. 10.1111/j.1574-6941.2012.01313.x 22273466

[B56] Jardine J. L. Abia A. L. K. Mavumengwana V. Ubomba-Jaswa E. (2017). Phylogenetic analysis and antimicrobial profiles of cultured emerging opportunistic pathogens (phyla *Actinobacteria* and *Proteobacteria*) identified in hot springs. *Int. J. Environ. Res. Public Health* 14 E1070. 10.3390/ijerph14091070 28914802 PMC5615607

[B57] Jiang X. Ellabaan M. M. H. Charusanti P. Munck C. Blin K. Tong Y. (2017). Dissemination of antibiotic resistance genes from antibiotic producers to pathogens. *Nat. Commun.* 8:15784. 10.1038/ncomms15784 28589945 PMC5467266

[B58] Ju F. Beck K. Yin X. Maccagnan A. McArdell C. S. Singer H. P. (2019). Wastewater treatment plant resistomes are shaped by bacterial composition, genetic exchange, and upregulated expression in the effluent microbiomes. *ISME J.* 13 346–360. 10.1038/s41396-018-0277-278 30250051 PMC6331547

[B59] Kai A. K. L. Cheung Y. K. Yeung P. K. K. Wong J. T. Y. (2006). Development of single-cell PCR methods for the Raphidophyceae. *Harmful Algae* 6 649–657. 10.1016/j.hal.2006.01.002

[B60] Knapp C. W. Callan A. C. Aitken B. Shearn R. Koenders A. Hinwood A. (2017). Relationship between antibiotic resistance genes and metals in residential soil samples from Western Australia. *Environ. Sci. Pollut. Res. Int.* 24 2484–2494. 10.1007/s11356-016-7997-y 27822686 PMC5340841

[B61] Kohanski M. A. DePristo M. A. Collins J. J. (2010). Sublethal antibiotic treatment leads to multidrug resistance via radical-induced mutagenesis. *Mol. Cell* 37 311–320. 10.1016/j.molcel.2010.01.003 20159551 PMC2840266

[B62] Krumperman P. H. (1983). Multiple antibiotic resistance indexing of *Escherichia coli* to identify high-risk sources of fecal contamination of foods. *Appl. Environ. Microbiol.* 46 165–170. 6351743 10.1128/aem.46.1.165-170.1983 PMC239283

[B63] Laverman A. M. Cazier T. Yan C. Roose-Amsaleg C. Petit F. Garnier J. (2015). Exposure to vancomycin causes a shift in the microbial community structure without affecting nitrate reduction rates in river sediments. *Environ. Sci. Pollut. Res.* 22 13702–13709. 10.1007/s11356-015-4159-4156 25663374

[B64] Lekunberri I. Villagrasa M. Balcázar J. L. Borrego C. M. (2017). Contribution of bacteriophage and plasmid DNA to the mobilization of antibiotic resistance genes in a river receiving treated wastewater discharges. *Sci. Total Environ.* 601-602 206–209. 10.1016/j.scitotenv.2017.05.174 28551539

[B65] Liew S. M. Rajasekaram G. Puthucheary S. A. Chua K. H. (2019). Antimicrobial susceptibility and virulence genes of clinical and environmental isolates of *Pseudomonas aeruginosa*. *PeerJ* 7 e6217. 10.7717/peerj.6217 30697478 PMC6346980

[B66] Lin L. Yuan K. Liang X. Chen X. Zhao Z. Yang Y. (2015). Occurrences and distribution of sulfonamide and tetracycline resistance genes in the Yangtze River Estuary and nearby coastal area. *Mar. Pollut. Bull.* 100 304–310. 10.1016/j.marpolbul.2015.08.036 26349787

[B67] Linares J. F. Gustafsson I. Baquero F. Martinez J. L. (2006). Antibiotics as intermicrobial signaling agents instead of weapons. *Proc. Natl. Acad. Sci. U.S.A.* 103 19484–19489. 10.1073/pnas.0608949103 17148599 PMC1682013

[B68] Liu Y. Y. Wang Y. Walsh T. R. Yi L. X. Zhang R. Spencer J. (2016). Emergence of plasmid-mediated colistin resistance mechanism MCR-1 in animals and human beings in China: a microbiological and molecular biological study. *Lancet Infect. Dis.* 16 161–168. 10.1016/S1473-3099(15)00424-427 26603172

[B69] Loos R. Carvalho R. Comero S. António D. C. Ghiani M. Lettieri T. (2012). EU wide monitoring survey on waste water treatment plant effluents. *Environ.Pollut.* 157 561–568. 10.1016/j.watres.2013.08.024 24091184

[B70] Luczkiewicz A. Kotlarska E. Artichowicz W. Tarasewicz K. Fudala-Ksiazek S. (2015). Antimicrobial resistance of *Pseudomonas* spp. isolated from wastewater and wastewater-impacted marine coastal zone. *Environ. Sci. Pollut. Res. Int.* 22 19823–19834. 10.1007/s11356-015-5098-y 26286796 PMC4679113

[B71] Manaia C. M. Rocha J. Scaccia N. Marano R. Radu E. Biancullo F. (2018). Antibiotic resistance in wastewater treatment plants: Tackling the black box. *Environ. Int.* 115 312–324. 10.1016/j.envint.2018.03.044 29626693

[B72] Martínez J. L. (2017). Effect of antibiotics on bacterial populations: a multi-hierarchical selection process. *F1000Res* 6 51. 10.12688/f1000research.9685.1 28163908 PMC5247793

[B73] Martínez-Aguirre A. García-León M. (1997). Radioactive impact of phosphate ore processing in a wet marshland in southwestern Spain. *J. Environ. Radioact.* 34 45–57. 10.1016/0265-931X(96)00015-X

[B74] Matyar F. Gülnaz O. Guzeldag G. Mercimek H. A. Akturk S. Arkut A. (2014). Antibiotic and heavy metal resistance in Gram-negative bacteria isolated from the Seyhan Dam Lake and Seyhan River in Turkey. *Ann. Microbiol.* 64 1033–1040. 10.1007/s13213-013-0740-748

[B75] Michael G. B. Kadlec K. Sweeney M. T. Brzuszkiewicz E. Liesegang H. Daniel R. (2012). ICEPmu1, an integrative conjugative element (ICE) of *Pasteurella multocida*: structure and transfer. *J. Antimicrob. Chemother.* 67 91–100. 10.1093/jac/dkr411 22001176

[B76] Narciso-da-Rocha C. Manaia C. M. (2016). Multidrug resistance phenotypes are widespread over different bacterial taxonomic groups thriving in surface water. *Sci. Total Environ.* 56 1–9. 10.1016/j.scitotenv.2016.04.062 27131885

[B77] Neeno-Eckwall E. C. Kinkel L. L. Schottel J. L. (2001). Competition and antibiosis in the biological control of potato scab. *Can. J. Microbiol.* 47 332–340. 10.1139/w01-010 11358173

[B78] Nguyen C. C. Hugie C. N. Kile M. L. Navab-Daneshmand T. (2019). Association between heavy metals and antibiotic-resistant human pathogens in environmental reservoirs: a review. *Front. Environ. Sci. Eng.* 13:46 10.1007/s11783-019-1129-1120

[B79] Pal C. Asiani K. Arya S. Rensing C. Stekel D. J. Larsson D. G. J. (2017). Metal resistance and its association with antibiotic resistance. *Adv. Microb. Physiol.* 70 261–313. 10.1016/bs.ampbs.2017.02.001 28528649

[B80] Pal C. Bengtsson-Palme J. Kristiansson E. Larsson D. G. (2015). Co-occurrence of resistance genes to antibiotics, biocides and metals reveals novel insights into their co-selection potential. *BMC Genomics* 16:964. 10.1186/s12864-015-2153-2155 26576951 PMC4650350

[B81] Papaslioti E.-M. Pérez-López R. Parviainen A. Macías F. Delgado-Huertas A. Garrido C. J. (2018). Stable isotope insights into the weathering processes of a phosphogypsum disposal area. *Water Res.* 140 344–353. 10.1016/j.watres.2018.04.060 29751316

[B82] Pasari N. Gupta M. Eqbal D. Yazdani S. S. (2019). Genome analysis of *Paenibacillus polymyxa* A18 gives insights into the features associated with its adaptation to the termite gut environment. *Sci. Rep.* 9 6091. 10.1038/s41598-019-42572-42575 30988376 PMC6465253

[B83] Pawlowski A. C. Wang W. Koteva K. Barton H. A. McArthur A. G. Wright G. D. (2016). A diverse intrinsic antibiotic resistome from a cave bacterium. *Nat. Commun.* 7:13803. 10.1038/ncomms13803 27929110 PMC5155152

[B84] Pawlowski A. C. Westman E. L. Koteva K. Waglechner N. Wright G. D. (2018). The complex resistomes of Paenibacillaceae reflect diverse antibiotic chemical ecologies. *ISME J.* 12 885–897. 10.1038/s41396-017-0017-15 29259290 PMC5864230

[B85] Pednekar P. B. Jain R. Thakur N. L. Mahajan G. B. (2010). Isolation of multi-drug resistant *Paenibacillus* sp. from fertile soil: an imminent menace of spreading resistance. *J. Life Sci.* 4 7–12.

[B86] Pérez-López R. Nieto J. M. López-Cascajosa M. J. Díaz-Blanco M. J. Sarmiento A. M. Oliveira V. (2011). Evaluation of heavy metals and arsenic speciation discharged by the industrial activity on the Tinto-Odiel estuary. *SW Spain. Mar. Pollut. Bull.* 62 405–411. 10.1016/j.marpolbul.2010.12.013 21215977

[B87] Perry J. Waglechner N. Wright G. (2016). The prehistory of antibiotic resistance. *Cold Spring Harb. Perspect. Med.* 6 a025197. 10.1101/cshperspect.a025197 27252395 PMC4888810

[B88] Poirel L. Potron A. Nordmann P. (2012). OXA-48-like carbapenemases: the phantom menace. *J. Antimicrob. Chemother.* 67 1597–1606. 10.1093/jac/dks121 22499996

[B89] Poirel L. Rodriguez-Martinez J. M. Mammeri H. Liard A. Nordmann P. (2005). Origin of plasmid-mediated quinolone resistance determinant QnrA. *Antimicrob. Agents Chemother.* 49 3523–3525. 10.1128/AAC.49.8.3523-3525.2005 16048974 PMC1196254

[B90] Poole K. (2017). At the nexus of antibiotics and metals: the impact of Cu and Zn on antibiotic activity and resistance. *Trends Microbiol.* 25 820–832. 10.1016/j.tim.2017.04.010 28526548

[B91] Prado N. Ochoa J. Amrane A. (2009). Biodegradation and biosorption of tetracycline and tylosin antibiotics in activated sludge system. *Process Biochem.* 44 1302–1306. 10.1016/j.procbio.2009.08.006

[B92] Proia L. Lupini G. Osorio V. Pérez S. Barceló D. Schwartz T. (2013). Response of biofilm bacterial communities to antibiotic pollutants in a Mediterranean river. *Chemosphere* 92 1126–1135. 10.1016/j.chemosphere.2013.01.063 23434260

[B93] Ramsay J. P. Firth N. (2017). Diverse mobilization strategies facilitate transfer of non-conjugative mobile genetic elements. *Curr. Opin. Microbiol.* 38 1–9. 10.1016/j.mib.2017.03.003 28391142

[B94] Rodgers K. McLellan I. Peshkur T. Williams R. Tonner R. Hursthouse A. S. (2019). Can the legacy of industrial pollution influence antimicrobial resistance in estuarine sediments? *Environ. Chem. Lett.* 17 595–607. 10.1007/s10311-018-0791-y

[B95] Rodríguez M. M. Power P. Radice M. Vay C. Famiglietti A. Galleni M. (2004). Chromosome-encoded CTX-M-3 from *Kluyvera ascorbata*: a possible origin of plasmid-borne CTX-M-1-derived cefotaximases. *Antimicrob. Agents Chemother.* 48 4895–4897. 10.1128/AAC.48.12.4895-4897.2004 15561876 PMC529199

[B96] Sáez-Niero J. A. Medina-Pascual M. J. Carrasco G. Garrido N. Fernandez-Torres M. A. Villalón P. (2017). Paenibacillus spp. isolated from human and environmental samples in Spain: detection of 11 new species. *New Microbes New Infect.* 19 19–27. 10.1016/j.nmni.2017.05.006 28702198 PMC5484988

[B97] Sainz A. Grande J. A. de la Torre M. L. (2004). Characterisation of heavy metal discharge into the Ria of Huelva. *Environ. Int.* 30 557–566. 10.1016/j.envint.2003.10.013 15031016

[B98] Sanseverino I. Navarro Cuenca A. Loos R. Marinov D. Lettieri T. (2018). *State of the Art on the Contribution of Water to Antimicrobial Resistance.* Brussels: European Union.

[B99] Selje N. Brinkhoff T. Simon M. (2005). Detection of abundant bacteria in the Weser Estuary using culture-dependent and culture-independent approaches. *Aquat. Microb. Ecol.* 39 17–34. 10.3354/ame039017

[B100] Sengupta S. Chattopadhyay M. K. Grossart H. P. (2013). The multifaceted roles of antibiotics and antibiotic resistance in nature. *Front. Microbiol.* 4:47 10.3389/fmicb.2013.00047 PMC3594987 23487476

[B101] Shen Y. Zhao W. Zhang C. Shi Y. S. (2017). Degradation of streptomycin in aquatic environment: kinetics, pathway, and antibacterial activity analysis. *Environ. Sci. Pollut. Res.* 24:14337. 10.1007/s11356-017-8978-8975 28429270

[B102] Sjöstedt J. Martiny J. B. H. Munk P. Riemann L. (2014). Abundance of broad bacterial taxa in the Sargasso Sea explained by environmental conditions but not water mass. *Appl. Environ. Microbiol.* 80 2786–2795. 10.1128/AEM.00099-14 24561593 PMC3993294

[B103] Soundarapandian P. Reena Singh P. S. Sowmiya S. (2013). Recombination of plasmid-borne drug resistant *Paenibacillus* sp. isolated from Crab (*Portunus sanguinolentus*). *Open Access Sci. Rep.* 2:590 10.4172/scientificreports.590

[B104] Spagnolo F. Rinaldi C. Sajorda D. R. Dykhuizen D. E. (2015). Evolution of resistance to continuously increasing streptomycin concentrations in populations of *Escherichia coli*. *Antimicrob. Agents Chemother.* 60 1336–1342. 10.1128/AAC.01359-1315 26666944 PMC4775953

[B105] Stefani F. O. Bell T. H. Marchand C. de la Providencia I. E. El Yassimi A. St-Arnaud M. (2015). Culture-dependent and -independent methods capture different microbial community fractions in hydrocarbon-contaminated soils. *PLoS One* 10:e0128272. 10.1371/journal.pone.0128272 26053848 PMC4460130

[B106] Sundin G. W. Bender C. L. (1996). Molecular analysis of closely related copper- and streptomycin-resistance plasmids in *Pseudomonas syringae* pv. syringae. *Plasmid* 35 98–107. 10.1006/plas.1996.0012 8700971

[B107] Suzuki S. Pruden A. Virta M. Zhang T. (2017). Editorial: antibiotic resistance in aquatic systems. *Front. Microbiol.* 8:14 10.3389/fmicb.2017.00014 PMC5263135 28179896

[B108] Tran N. H. Chen H. Reinhard M. Mao F. Gin K. Y.-H. (2016). Occurrence and removal of multiple classes of antibiotics and antimicrobial agents in biological wastewater treatment processes. *Water Res.* 104 461–472. 10.1016/j.watres.2016.08.040 27585426

[B109] Tripathi V. Cytryn E. (2017). Impact of anthropogenic activities on the dissemination of antibiotic resistance across ecological boundaries. *Essays Biochem.* 61 11–21. 10.1042/EBC20160054 28258226

[B110] Vaz-Moreira I. Nunes O. C. Manaia C. M. (2012). Diversity and antibiotic resistance in *Pseudomonas* spp. from drinking water. *Sci. Total Environ.* 426 366–374. 10.1016/j.scitotenv.2012.03.046 22521167

[B111] Vaz-Moreira I. Nunes O. C. Manaia C. M. (2014). Bacterial diversity and antibiotic resistance in water habitats: searching the links with the human microbiome. *FEMS Microbiol. Rev.* 38 761–778. 10.1111/1574-6976.12062 24484530

[B112] Voolaid V. Jõers A. Kisand V. Tenson T. (2012). Co-occurrence of resistance to different antibiotics among aquatic bacteria. *BMC Microbiol.* 12:225. 10.1186/1471-2180-12-225 23031674 PMC3519559

[B113] Walsh F. Duffy B. (2013). The culturable soil antibiotic resistome: a community of multi-drug resistant bacteria. *PLoS One* 8:e65567. 10.1371/journal.pone.0065567 23776501 PMC3680443

[B114] Wang X. Yang F. Zhao J. Xu Y. Mao D. Zhu X. (2018). Bacterial exposure to ZnO nanoparticles facilitates horizontal transfer of antibiotic resistance genes. *NanoImpact* 10 61–67. 10.1016/j.impact.2017.11.006

[B115] Wang Y. Zhang H. Zhang J. Lu C. Huang Q. Wu J. (2011). Degradation of tetracycline in aqueous media by ozonation in an internal loop-lift reactor. *J. Hazard Mat.* 192 35–43. 10.1016/j.jhazmat.2011.04.086 21616591

[B116] Weisburg W. G. Barns S. M. Pelletier D. A. Lane D. J. (1991). 16S ribosomal DNA amplification for phylogenetic study. *J. Bacteriol.* 173 697–703. 10.1128/jb.173.2.697-703.1991 1987160 PMC207061

[B117] Wilkinson J. Boxall A. (2019). “The first global study of pharmaceutical contamination in riverine environments. Communication 339,” in *SETAC Europe 29th Annual Meeting* (Helsinki).

[B118] World Health Organization [WHO] (2018). *Global Antimicrobial Resistance Surveillance System (GLASS) Report: early Implementation 2016-2017.* Geneva: WHO.

[B119] Xie J. Shi H. Du Z. Wang T. Liu X. Chen S. (2016). Comparative genomic and functional analysis reveal conservation of plant growth promoting traits in *Paenibacillus polymyxa* and its closely related species. *Sci. Rep.* 6:21329. 10.1038/srep21329 26856413 PMC4746698

[B120] Xiong L. Liao D. Lu X. Yan H. Shi L. Mo Z. (2017). Proteomic analysis reveals that a global response is induced by subinhibitory concentrations of ampicillin. *Bioengineered* 7 1–10. 10.1080/21655979.2017.1373532 28881168 PMC5736336

[B121] Xu H. Qin S. Lan Y. Liu M. Cao X. Qiao D. (2017). Comparative genomic analysis of *Paenibacillus* sp. *SSG-*1 and its closely related strains reveals the effect of glycometabolism on environmental adaptation. *Sci. Rep.* 7:5720. 10.1038/s41598-017-06160-6169 28720902 PMC5516027

[B122] Yang J. Wang C. Shu C. Liu L. Geng J. Hu S. (2013). Marine sediment bacteria harbor antibiotic resistance genes highly similar to those found in human pathogens. *Microb. Ecol.* 65 975–981. 10.1007/s00248-013-0187-182 23370726

[B123] Yang Y. Li B. Ju F. Zhang T. (2013). Exploring variation of antibiotic resistance genes in activated sludge over a four-year period through a metagenomic approach. *Environ. Sci. Technol.* 47 10197–10205. 10.1021/es4017365 23919449

[B124] Ye W. J. Liu X. L. Lin S. Q. Tan J. Pan J. L. Li D. T. (2009). The vertical distribution of bacterial and archaeal communities in the water and sediment of Lake Taihu. *FEMS Microbiol. Ecol.* 70 263–276. 10.1111/j.1574-6941.2009.00761.x 19744240

[B125] Yim G. Wang H. H. Davies J. (2007). Antibiotics as signalling molecules. *Philos. Trans. R. Soc. Lond. B Biol. Sci.* 362 1195–1200. 10.1098/rstb.2007.2044 17360275 PMC2435582

[B126] Zhang Q. Q. Tian G. M. Jin R. C. (2018). The occurrence, maintenance, and proliferation of antibiotic resistance genes (ARGs) in the environment: influencing factors, mechanisms, and elimination strategies. *Appl. Microbiol. Biotechnol.* 102 8261–8274. 10.1007/s00253-018-9235-9237 30056512

[B127] Zhang X. X. Zhang T. (2011). Occurrence, abundance, and diversity of tetracycline resistance genes in 15 sewage treatment plants across China and other global locations. *Environ. Sci. Technol.* 45 2598–2604. 10.1021/es103672x 21388174

[B128] Zhao P. Xue Y. Gao W. Li J. Zu X. Fu D. (2018). Bacillaceae-derived peptide antibiotics since 2000. *Peptides* 101 10–16. 10.1016/j.peptides.2017.12.018 29269072

[B129] Zhao Y. Cocerva T. Cox S. Tardif S. Su J.-Q. Zhu Y.-G. (2019). Evidence for co-selection of antibiotic resistance genes and mobile genetic elements in metal polluted urban soils. *Sci. Total Environ.* 656 512–520. 10.1016/j.scitotenv.2018.11.372 30529954

[B130] Zhou L. J. Ying G. G. Liu S. Zhao J. L. Yang B. Chen Z. F. (2013). Occurrence and fate of eleven classes of antibiotics in two typical wastewater treatment plants in South China. *Sci. Total Environ.* 45 365–376. 10.1016/j.scitotenv.2013.03.010 23538107

