# Supplementary figures and images for: Bacteria From the Multi-Contaminated Tinto River Estuary (SW, Spain) Show High Multi-Resistance to Antibiotics and Point to Paenibacillus spp. as Antibiotic-Resistance-Dissemination Players
Source: Front Microbiol. 2020 Jan 10;10:3071. doi: 10.3389/fmicb.2019.03071 (PMC6965355; doi:10.3389/fmicb.2019.03071)

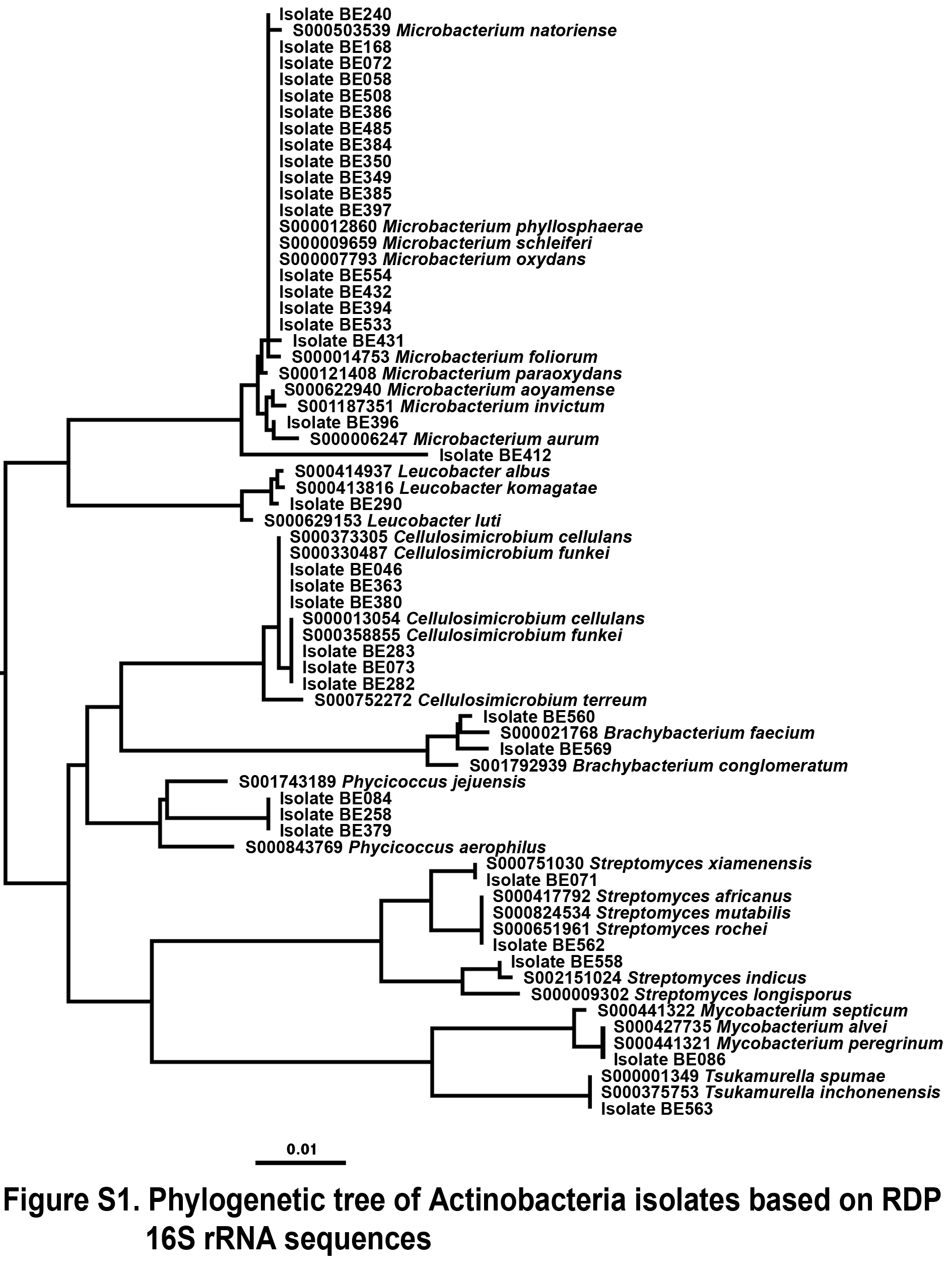

Supplement: Supplementary file 1 [file Image_1.TIF]

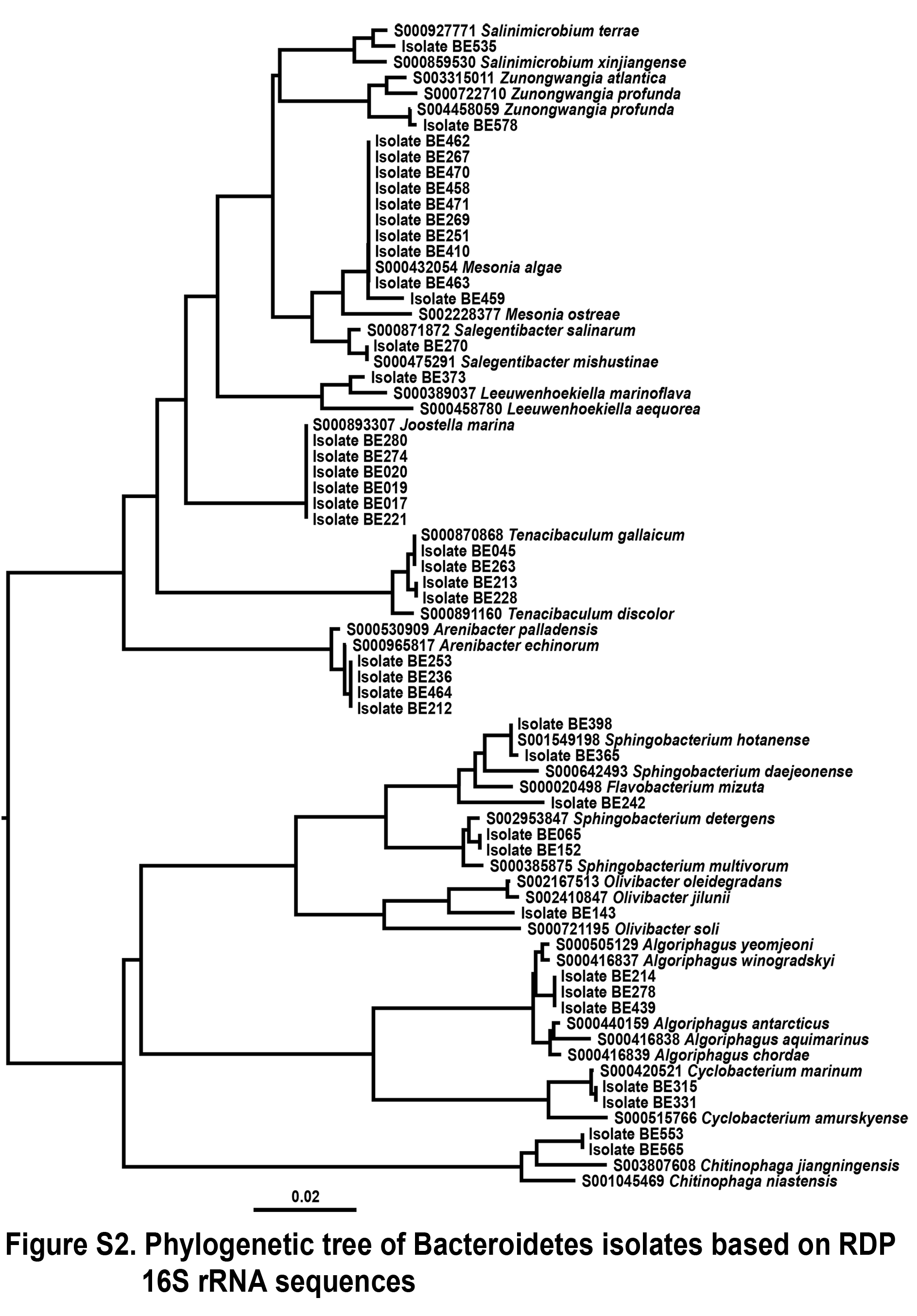

Supplement: Supplementary file 2 [file Image_2.TIF]

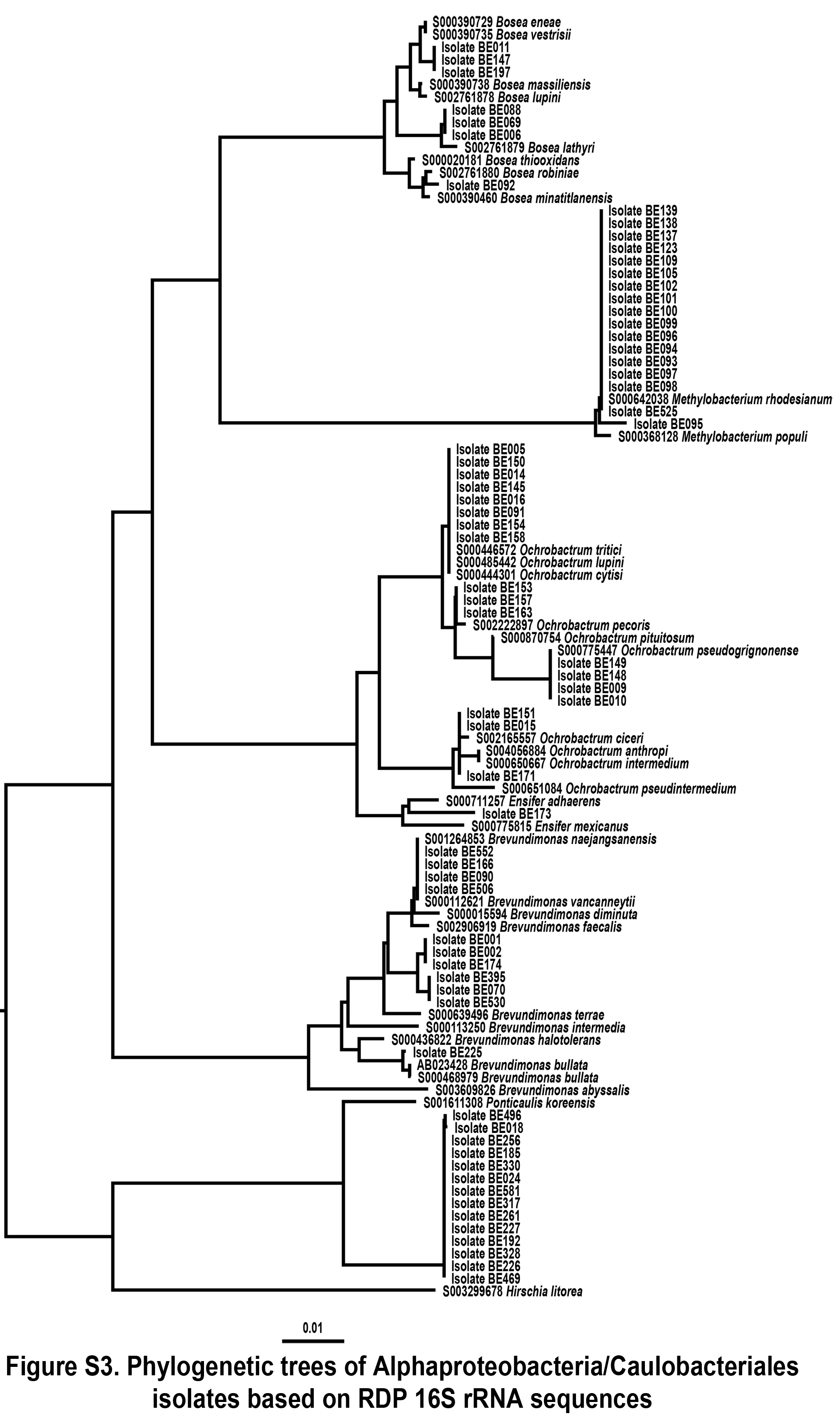

Supplement: Supplementary file 3 [file Image_3.TIF]

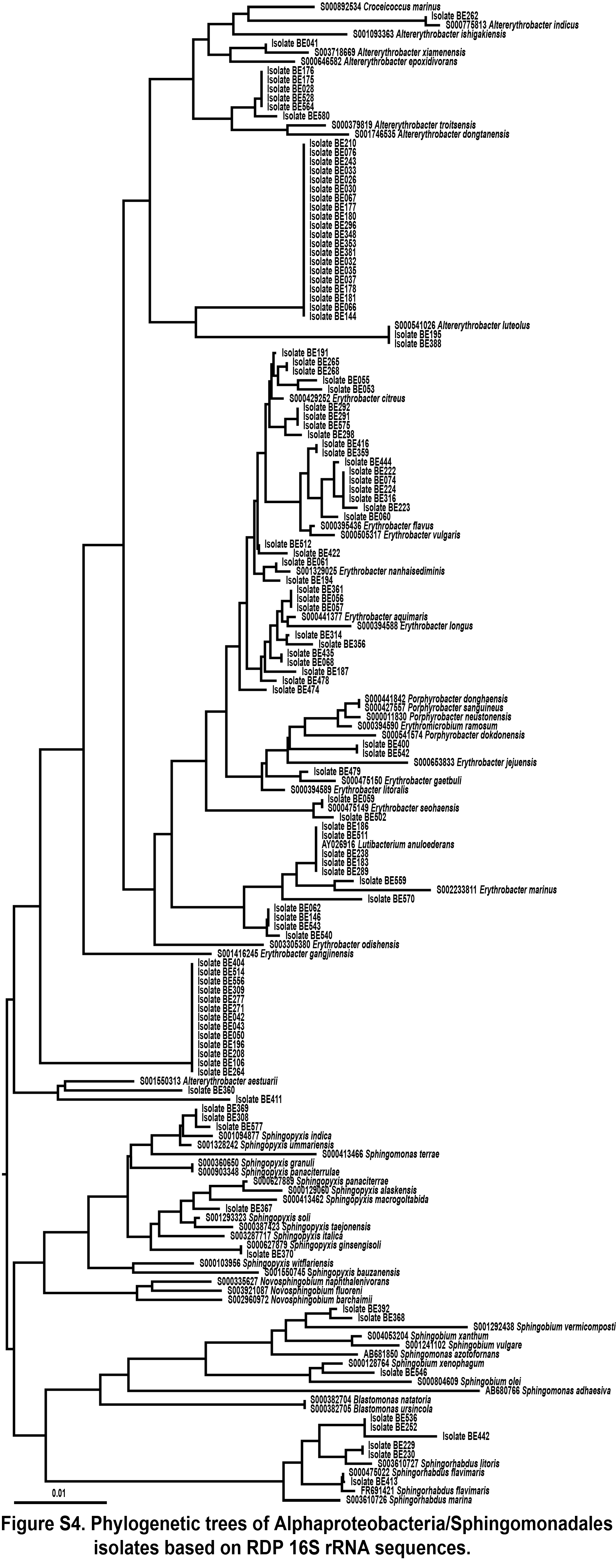

Supplement: Supplementary file 4 [file Image_4.TIF]

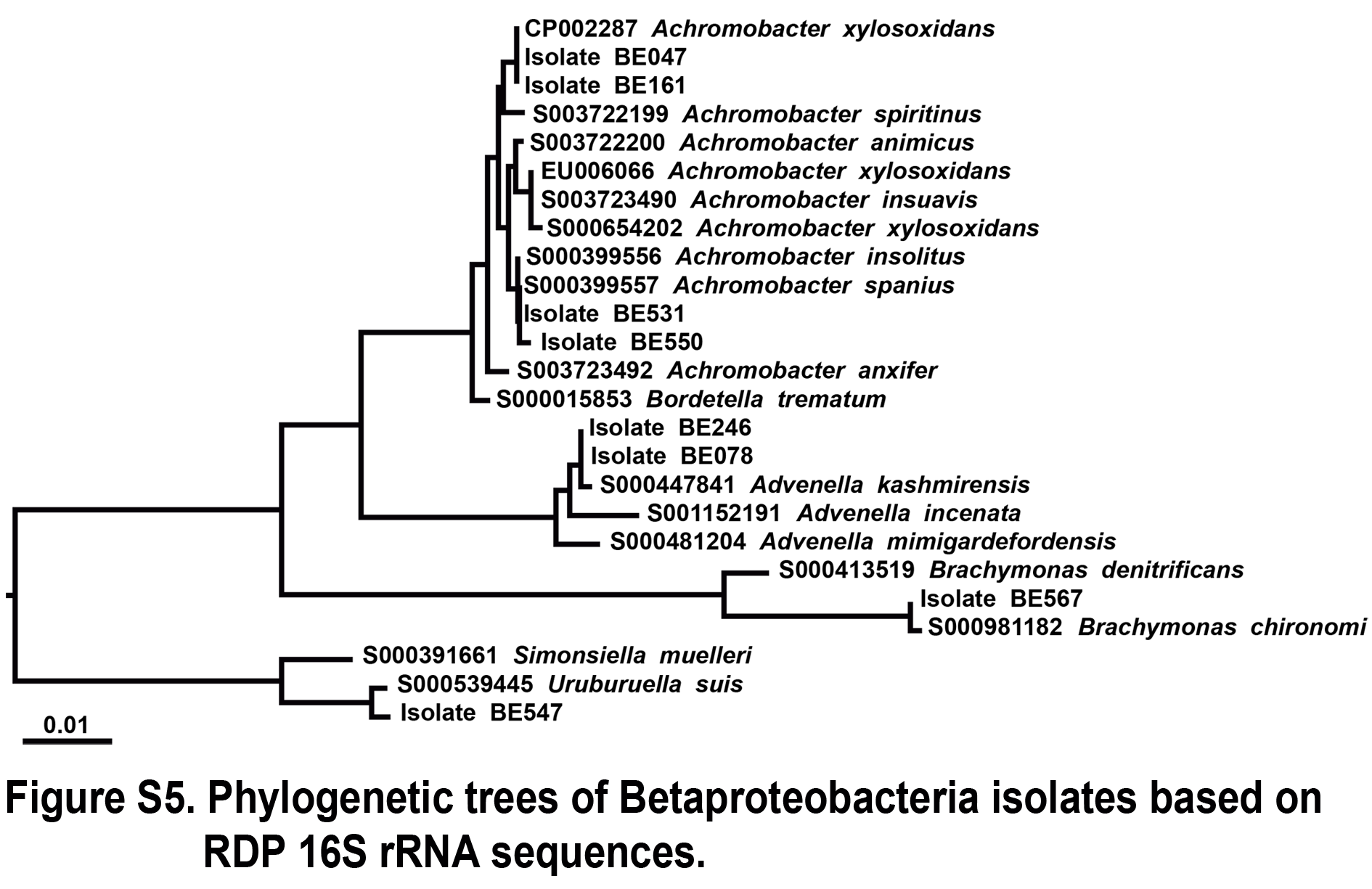

Supplement: Supplementary file 5 [file Image_5.TIF]

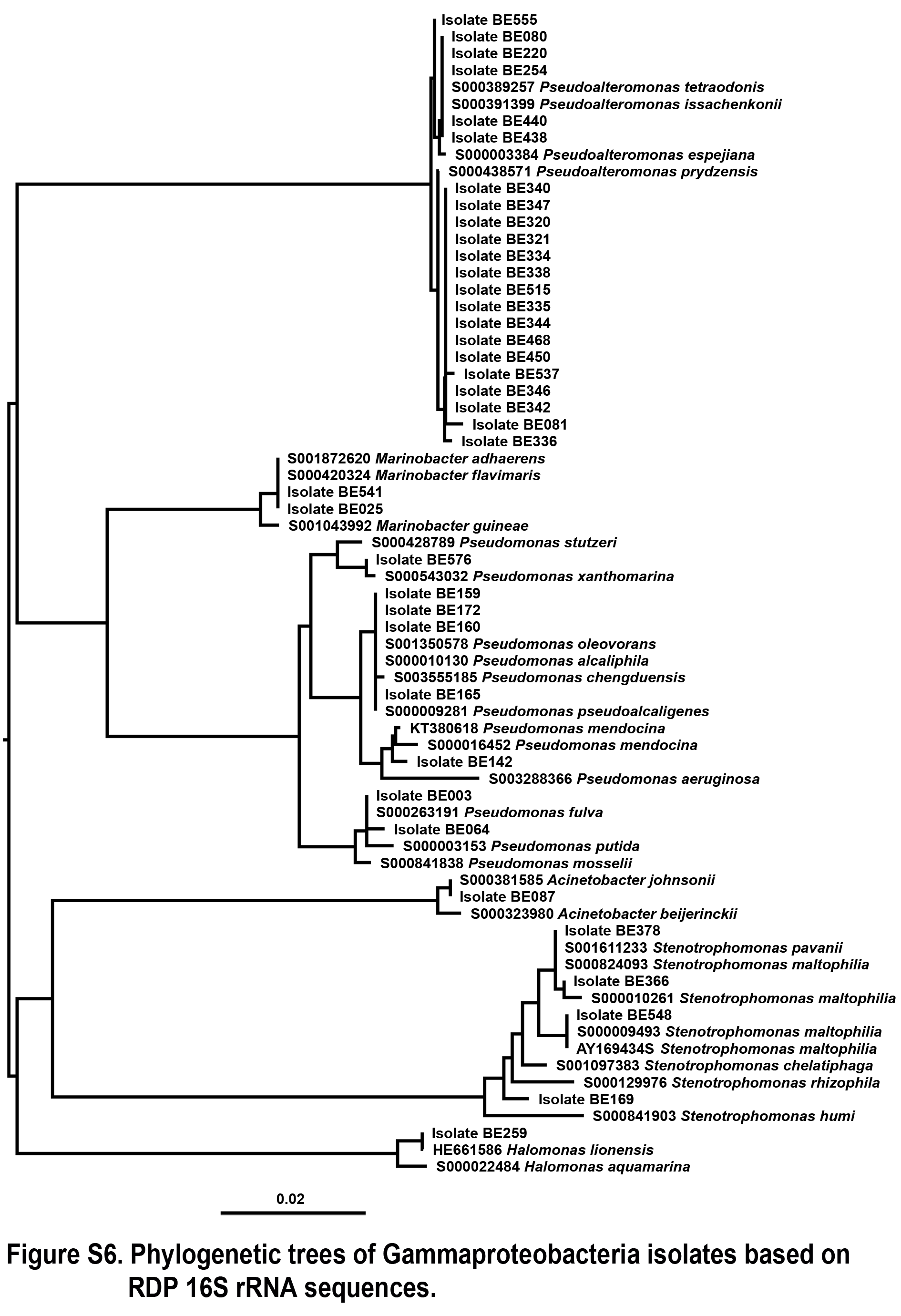

Supplement: Supplementary file 6 [file Image_6.TIF]

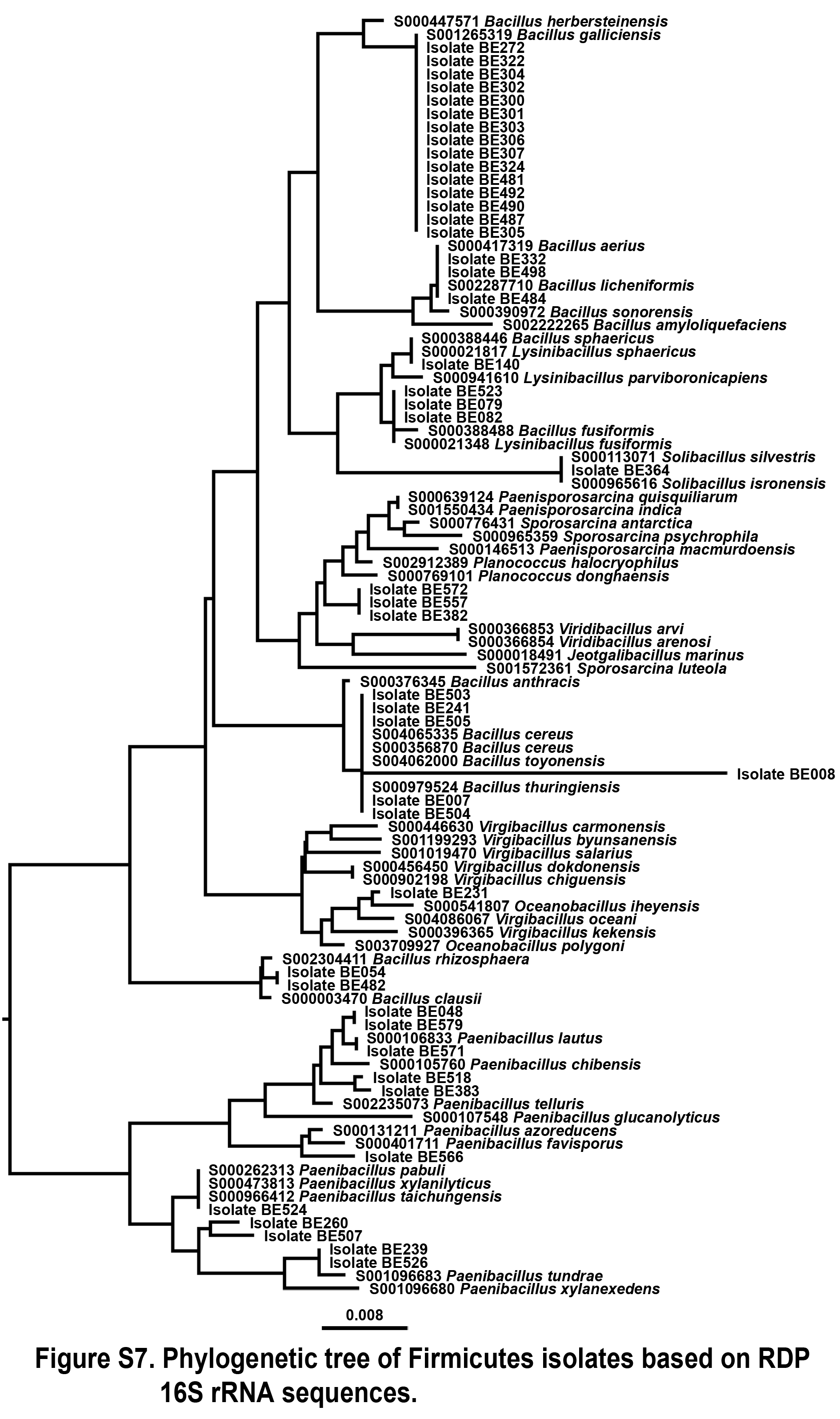

Supplement: Supplementary file 7 [file Image_7.TIF]
