# Supplementary material for: Bacteria From the Multi-Contaminated Tinto River Estuary (SW, Spain) Show High Multi-Resistance to Antibiotics and Point to Paenibacillus spp. as Antibiotic-Resistance-Dissemination Players
Source: Front Microbiol. 2020 Jan 10;10:3071. doi: 10.3389/fmicb.2019.03071 (PMC6965355; doi:10.3389/fmicb.2019.03071)
Supplement: Supplementary file 8 [file Table_1.DOCX]

| Sample | pH | T (ºC) | Salinity | Conductivity  (mS/cm) | Redox potential  (mV) |
| --- | --- | --- | --- | --- | --- |
| H1L | **6.2** | **17.5** | **27.1** | **42.1** | **159** |
| H2-I | **6.5** | **20.6** | **31.1** | **47.8** | **178** |
| H2-II | **6.3** | **19.4** | **31.9** | **48.5** | **183** |
| H2-III | **6.4** | **18.8** | **31.7** | **48.6** | **208** |

**Table S1. Physical parameters of water samples**
