# Supplementary material for: Bacteria From the Multi-Contaminated Tinto River Estuary (SW, Spain) Show High Multi-Resistance to Antibiotics and Point to Paenibacillus spp. as Antibiotic-Resistance-Dissemination Players
Source: Front Microbiol. 2020 Jan 10;10:3071. doi: 10.3389/fmicb.2019.03071 (PMC6965355; doi:10.3389/fmicb.2019.03071)
Supplement: Supplementary file 9 [file Table_2.DOCX]

|  | H1L (µg/L) | H2L (µg/L) |
| --- | --- | --- |
| As | **15.9** | **6.2** |
| Au | **3.9** | **4.2** |
| B | **5232.7** | **6198.9** |
| Ba | **30.9** | **30.0** |
| Be | **10.4** | **Not det.** |
| Bi | **5.4** | **5.7** |
| Ca | **452126.5** | **504920.0** |
| Co | **0.4** | **1.2** |
| Cr | **59.0** | **29.5** |
| Cu | **74.2** | **20.9** |
| Fe | **5974.2** | **1686.9** |
| K | **332200.2** | **392848.9** |
| Li | **97.8** | **212.7** |
| Mg | **1171845.7** | **1419220.2** |
| Mn | **91.3** | **73.3** |
| Na | **8989404.6** | **10858108.9** |
| Ni | **29.2** | **13.2** |
| Pb | **8.0** | **8.8** |
| Rb | **7.4** | **126.0** |
| Sr | **6369.7** | **7600.3** |
| Th | **28.5** | **26.7** |
| U | **35.2** | **38.3** |
| V | **121.7** | **145.1** |
| Y | **0.3** | **0.2** |

**Table S2. Elemental composition of water samples.**
