# Supplementary material for: Bacteria From the Multi-Contaminated Tinto River Estuary (SW, Spain) Show High Multi-Resistance to Antibiotics and Point to Paenibacillus spp. as Antibiotic-Resistance-Dissemination Players
Source: Front Microbiol. 2020 Jan 10;10:3071. doi: 10.3389/fmicb.2019.03071 (PMC6965355; doi:10.3389/fmicb.2019.03071)
Supplement: Supplementary file 10 [file Table_3.DOCX]

|  | H1S (mg/g) | H2S (mg/g) |
| --- | --- | --- |
| As | **0.602±0.003** | **1.135±0.005** |
| Br | **0.014±0.001** | **0.006±0.001** |
| Ca | **5.690±0.036** | **11.560±0.058** |
| Cl | **122.053±0.270** | **67.507±0.249** |
| Cr | **0.058±0.007** | **0.053±0.007** |
| Cu | **2.180±0.007** | **0.485±0.004** |
| Fe | **59.328±0.050** | **38.607±0.049** |
| K | **5.317±0.039** | **3.231±0.039** |
| Mn | **0.138±0.006** | **0.197±0.007** |
| Ni | **Not det.** | **Not det.** |
| P | **8.786±0.227** | **4.141±0.220** |
| Rb | **0.153±0.001** | **0.114±0.001** |
| S | **4.929±0.107** | **1.634±0.107** |
| Sr | **0.137±0.001** | **0.175±0.002** |
| Ti | **0.998±0.013** | **0.842±0.014** |
| Zn | **1.256±0.005** | **1.411±0.006** |

**Table S3. Elemental composition of sediment samples.**
