# Supplementary material for: Bacteria From the Multi-Contaminated Tinto River Estuary (SW, Spain) Show High Multi-Resistance to Antibiotics and Point to Paenibacillus spp. as Antibiotic-Resistance-Dissemination Players
Source: Front Microbiol. 2020 Jan 10;10:3071. doi: 10.3389/fmicb.2019.03071 (PMC6965355; doi:10.3389/fmicb.2019.03071)
Supplement: Supplementary file 11 [file Table_4.DOCX]

| ANTIBIOTIC RESISTANCE PROFILES | Profile No. | NUMBER OF ISOLATES | | | | | | | |
| --- | --- | --- | --- | --- | --- | --- | --- | --- | --- |
|  |  | **H1L-M** | **H1S-M** | **H2L-M** | **H2S-M** | **H1L-N** | **H1S-N** | **H2L-N** | **H2S-N** |
| Ap | **1.01** | **5** | **1** | **4** | **2** | **0** | **0** | **0** | **0** |
| Ap, Cc | **2.01** | **0** | **0** | **1** | **0** | **0** | **0** | **0** | **0** |
| Ap, Sm | **2.02** | **0** | **0** | **2** | **0** | **0** | **0** | **1** | **0** |
| Ap, Tc | **2.03** | **2** | **0** | **0** | **1** | **0** | **0** | **0** | **0** |
| Ap, Tm | **2.04** | **0** | **0** | **0** | **0** | **0** | **0** | **1** | **0** |
| Ap, Vm | **2.05** | **0** | **0** | **0** | **0** | **0** | **1** | **0** | **0** |
| Ap, Cc, Em | **3.01** | **0** | **0** | **0** | **0** | **0** | **1** | **0** | **0** |
| Ap, Cz, Km | **3.02** | **0** | **0** | **0** | **0** | **0** | **0** | **1** | **0** |
| Ap, Cz, Sm | **3.03** | **0** | **0** | **0** | **0** | **0** | **0** | **1** | **0** |
| Ap, Cz, Tc | **3.04** | **0** | **0** | **1** | **0** | **0** | **0** | **0** | **0** |
| Ap, Cz, Tm | **3.05** | **0** | **0** | **0** | **0** | **2** | **2** | **4** | **0** |
| Ap, Em, Sm | **3.06** | **0** | **0** | **0** | **0** | **1** | **0** | **0** | **0** |
| Ap, Km, Sm | **3.07** | **0** | **0** | **1** | **0** | **0** | **0** | **0** | **0** |
| Ap, Nx, Sm | **3.08** | **0** | **0** | **1** | **0** | **0** | **0** | **0** | **0** |
| Ap, Nx,Tm | **3.09** | **0** | **0** | **0** | **0** | **0** | **0** | **0** | **1** |
| Ap, Sm, Tc | **3.10** | **1** | **0** | **0** | **0** | **0** | **0** | **0** | **0** |
| Ap, Tc, Tm | **3.11** | **1** | **1** | **0** | **0** | **0** | **0** | **0** | **0** |
| Ap, Tm, Vm | **3.12** | **0** | **0** | **0** | **0** | **1** | **0** | **0** | **0** |
| Ap, Cz, Em, Tm | **4.01** | **0** | **0** | **0** | **0** | **0** | **0** | **1** | **0** |
| Ap, Cz, Km, Sm | **4.02** | **0** | **0** | **1** | **0** | **0** | **0** | **0** | **0** |
| Ap, Cz, Km, Tc | **4.03** | **0** | **1** | **0** | **0** | **0** | **0** | **0** | **0** |
| Ap, Cz, Nx, Sm | **4.04** | **0** | **1** | **0** | **0** | **0** | **0** | **0** | **0** |
| Ap, Cz, Nx, Tm | **4.05** | **0** | **0** | **0** | **0** | **1** | **0** | **0** | **0** |
| Ap, Cz, Sm, Tc | **4.06** | **0** | **0** | **2** | **0** | **0** | **0** | **0** | **0** |
| Ap, Cz, Sm, Tm | **4.07** | **0** | **0** | **0** | **0** | **1** | **0** | **1** | **1** |
| Ap, Em, Nx, Sm | **4.08** | **0** | **0** | **0** | **0** | **0** | **1** | **0** | **0** |
| Ap, Em, Rp, Sm | **4.09** | **0** | **0** | **0** | **0** | **0** | **0** | **1** | **0** |
| Ap, Km, Nx, Tc | **4.10** | **0** | **0** | **1** | **0** | **0** | **0** | **0** | **0** |
| Ap, Km, Tc, Tm | **4.11** | **0** | **0** | **1** | **0** | **0** | **0** | **0** | **0** |
| Ap, Nx, Sm, Tc | **4.12** | **6** | **6** | **16** | **1** | **0** | **0** | **0** | **0** |
| Ap, Nx, Sm, Tm | **4.13** | **1** | **0** | **1** | **0** | **0** | **0** | **0** | **0** |
| Ap, Nx, Tc, Tm | **4.14** | **1** | **0** | **0** | **0** | **0** | **0** | **0** | **0** |
| Ap, Cz, Em, Nx, Sm | **5.01** | **2** | **0** | **0** | **0** | **0** | **0** | **0** | **0** |
| Ap, Cz, Km, Nx, Sm | **5.02** | **0** | **0** | **0** | **0** | **0** | **1** | **0** | **0** |
| Ap, Cz, Km, Sm, Tc | **5.03** | **0** | **0** | **3** | **0** | **0** | **0** | **0** | **0** |
| Ap, Cz, Km, Tc, Tm | **5.04** | **1** | **0** | **0** | **0** | **0** | **0** | **0** | **0** |
| Ap, Cz, Nx, Sm, Tc | **5.05** | **1** | **1** | **1** | **0** | **0** | **0** | **0** | **0** |
| Ap, Cz, Nx, Sm, Tm | **5.06** | **2** | **0** | **0** | **3** | **1** | **1** | **0** | **2** |
| Ap, Cz, Nx, Tc, Tm | **5.07** | **3** | **0** | **1** | **0** | **0** | **0** | **0** | **0** |
| Ap, Cz, Rp, Sm, Tm | **5.08** | **0** | **0** | **0** | **0** | **1** | **0** | **0** | **0** |
| Ap, Cz, Sm, Tc, Tm | **5.09** | **0** | **0** | **4** | **0** | **0** | **0** | **0** | **0** |
| Ap, Cz, Sm, Tm, Vm | **5.10** | **0** | **0** | **1** | **0** | **0** | **0** | **0** | **0** |
| Ap, Em, Rp, Sm, Tm | **5.11** | **0** | **0** | **0** | **0** | **1** | **0** | **0** | **1** |
| Ap, Km, Nx, Sm, Tc | **5.12** | **1** | **0** | **3** | **0** | **0** | **0** | **0** | **0** |
| Ap, Km, Nx, Tc, Tm | **5.13** | **1** | **0** | **0** | **0** | **0** | **0** | **0** | **0** |
| Ap, Nx, Rp, Sm, Tc | **5.14** | **2** | **0** | **1** | **0** | **0** | **0** | **0** | **0** |
| Ap, Nx, Sm, Tc, Tm | **5.15** | **17** | **1** | **33** | **1** | **0** | **0** | **0** | **0** |
| Ap, Nx, Sm, Tm, Vm | **5.16** | **0** | **0** | **0** | **0** | **1** | **0** | **0** | **0** |
| Ap, Nx, Tc, Tm, Vm | **5.17** | **0** | **0** | **1** | **0** | **0** | **0** | **0** | **0** |
| Ap, Sm, Tc, Tm, Vm | **5.18** | **1** | **0** | **0** | **0** | **0** | **0** | **0** | **0** |
| Ap, Cc, Cz, Em, Nx, Sm | **6.01** | **0** | **0** | **0** | **0** | **0** | **1** | **0** | **0** |
| Ap, Cc, Sm, Tc, Tm, Vm | **6.02** | **1** | **0** | **0** | **0** | **0** | **0** | **0** | **0** |
| Ap, Cz, Em, Km, Nx, Sm | **6.03** | **0** | **0** | **0** | **0** | **0** | **1** | **0** | **0** |
| Ap, Cz, Em, Km, Sm, Tm | **6.04** | **0** | **0** | **0** | **0** | **0** | **1** | **0** | **0** |
| Ap, Cz, Em, Nx, Sm, Tm | **6.05** | **0** | **0** | **0** | **0** | **3** | **0** | **0** | **1** |
| Ap, Cz, Em, Rp, Sm, Tm | **6.06** | **0** | **0** | **0** | **0** | **1** | **0** | **0** | **0** |
| Ap, Cz, Em, Sm, Tc, Tm | **6.07** | **0** | **0** | **1** | **0** | **0** | **0** | **0** | **0** |
| Ap, Cz, Km, Nx, Sm, Tc | **6.08** | **0** | **0** | **2** | **0** | **0** | **0** | **0** | **0** |
| Ap, Cz, Km, Nx, Tc, Tm | **6.09** | **0** | **0** | **0** | **0** | **0** | **0** | **1** | **0** |
| Ap, Cz, Km, Sm, Tc, Tm | **6.10** | **1** | **0** | **1** | **0** | **0** | **0** | **0** | **0** |
| Ap, Cz, Km, Sm, Tm, Vm | **6.11** | **1** | **0** | **0** | **0** | **0** | **0** | **0** | **0** |
| Ap, Cz, Nx, Rp, Sm, Tc | **6.12** | **1** | **0** | **0** | **0** | **0** | **0** | **0** | **0** |
| Ap, Cz, Nx, Rp, Sm, Tm | **6.13** | **0** | **0** | **0** | **0** | **1** | **1** | **0** | **0** |
| Ap, Cz, Nx, Sm, Tc, Tm | **6.14** | **26** | **9** | **10** | **6** | **0** | **0** | **0** | **0** |
| Ap, Cz, Nx, Sm, Tm, Vm | **6.15** | **0** | **0** | **0** | **0** | **2** | **2** | **0** | **0** |
| Ap, Cz, Sm, Tc, Tm, Vm | **6.16** | **0** | **0** | **1** | **0** | **0** | **0** | **0** | **0** |
| Ap, Em, Nx, Rp, Sm, Vm | **6.17** | **0** | **0** | **0** | **0** | **0** | **1** | **0** | **0** |
| Ap, Em, Nx, Sm, Tc, Tm | **6.18** | **0** | **0** | **0** | **0** | **0** | **1** | **0** | **0** |
| Ap, Em, Nx, Sm, Tm, Vm | **6.19** | **0** | **0** | **0** | **0** | **0** | **1** | **0** | **0** |
| Ap, Em, Rp, Sm, Tm, Vm | **6.20** | **0** | **0** | **0** | **0** | **0** | **0** | **1** | **0** |
| Ap, Km, Nx, Rp, Sm, Tc | **6.21** | **1** | **0** | **0** | **0** | **0** | **0** | **0** | **0** |
| Ap, Km, Nx, Sm, Tc, Tm | **6.22** | **5** | **0** | **6** | **0** | **0** | **0** | **0** | **0** |
| Ap, Km, Nx, Sm, Tc, Vm | **6.23** | **1** | **0** | **0** | **0** | **0** | **0** | **0** | **0** |
| Ap, Km, Sm, Tc, Tm, Vm | **6.24** | **1** | **0** | **0** | **0** | **0** | **0** | **0** | **0** |
| Ap, Nx, Rp, Sm, Tc, Tm | **6.25** | **1** | **0** | **3** | **1** | **0** | **0** | **0** | **0** |
| Ap, Rp, Sm, Tc, Tm, Vm | **6.26** | **0** | **0** | **1** | **0** | **0** | **0** | **0** | **0** |
| Ap, Cc, Cz, Em, Nx, Sm, Tm | **7.01** | **0** | **0** | **0** | **0** | **1** | **1** | **0** | **0** |
| Ap, Cc, Cz, Nx, Rp, Tc, Vm | **7.02** | **0** | **0** | **0** | **0** | **0** | **1** | **0** | **0** |

| Ap, Cc, Cz, Nx, Sm, Tc, Tm | 7.03 | 1 | 0 | 0 | 0 | 0 | 0 | 0 | 0 |
| --- | --- | --- | --- | --- | --- | --- | --- | --- | --- |
| Ap, Cc, Em, Nx, Sm, Tm, Vm | **7.04** | **0** | **0** | **0** | **0** | **0** | **1** | **0** | **0** |
| Ap, Cc, Km, Nx, Sm, Tc, Tm | **7.05** | **1** | **0** | **1** | **0** | **0** | **0** | **0** | **0** |
| Ap, Cc, Km, Nx, Tc, Tm, Vm | **7.06** | **1** | **0** | **0** | **0** | **0** | **0** | **0** | **0** |
| Ap, Cc, Nx, Rp, Sm, Tc, Tm | **7.07** | **1** | **0** | **1** | **0** | **0** | **0** | **0** | **0** |
| Ap, Cc, Nx, Rp, Tc, Tm, Vm | **7.08** | **0** | **0** | **0** | **0** | **1** | **0** | **0** | **0** |
| Ap, Cz, Em, Km, Nx, Sm, Tm | **7.09** | **0** | **0** | **0** | **1** | **0** | **1** | **0** | **0** |
| Ap, Cz, Em, Km, Rp, Tm, Vm | **7.10** | **0** | **0** | **0** | **0** | **0** | **2** | **0** | **0** |
| Ap, Cz, Em, Km, Tc, Tm, Vm | **7.11** | **0** | **0** | **0** | **0** | **1** | **0** | **0** | **0** |
| Ap, Cz, Em, Nx, Rp, Sm, Tm | **7.12** | **0** | **0** | **0** | **0** | **1** | **1** | **0** | **0** |
| Ap, Cz, Em, Nx, Sm, Tc, Tm | **7.13** | **0** | **0** | **1** | **0** | **0** | **0** | **0** | **0** |
| Ap, Cz, Em, Nx, Sm, Tm, Vm | **7.14** | **0** | **0** | **0** | **0** | **0** | **2** | **0** | **4** |
| Ap, Cz, Em, Rp, Sm, Tm, Vm | **7.15** | **0** | **0** | **0** | **0** | **0** | **1** | **0** | **0** |
| Ap, Cz, Km, Nx, Rp, Sm, Tm | **7.16** | **0** | **0** | **0** | **1** | **0** | **0** | **0** | **0** |
| Ap, Cz, Km, Nx, Sm, Tc, Tm | **7.17** | **4** | **7** | **11** | **3** | **0** | **0** | **0** | **0** |
| Ap, Cz, Km, Nx, Sm, Tc, Vm | **7.18** | **0** | **0** | **0** | **1** | **0** | **0** | **0** | **0** |
| Ap, Cz, Km, Nx, Tc, Tm, Vm | **7.19** | **0** | **0** | **3** | **1** | **0** | **0** | **0** | **0** |
| Ap, Cz, Km, Sm, Tc, Tm, Vm | **7.20** | **5** | **1** | **2** | **0** | **0** | **0** | **0** | **0** |
| Ap, Cz, Nx, Rp, Sm, Tc, Tm | **7.21** | **6** | **4** | **2** | **3** | **0** | **0** | **0** | **0** |
| Ap, Cz, Nx, Rp, Sm, Tm, Vm | **7.22** | **0** | **0** | **0** | **0** | **0** | **0** | **0** | **1** |
| Ap, Cz, Nx, Sm, Tc, Tm, Vm | **7.23** | **1** | **0** | **1** | **0** | **0** | **0** | **0** | **0** |
| Ap, Em, Km, Nx, Rp, Sm, Tm | **7.24** | **0** | **0** | **0** | **0** | **1** | **0** | **1** | **0** |
| Ap, Em, Km, Nx, Sm, Tc, Tm | **7.25** | **1** | **0** | **1** | **0** | **0** | **0** | **0** | **0** |
| Ap, Km, Nx, Rp, Sm, Tc, Tm | **7.26** | **1** | **0** | **2** | **0** | **0** | **0** | **0** | **0** |
| Ap, Cc, Cz, Em, Km, Nx, Sm, Tm | **8.01** | **0** | **0** | **0** | **0** | **0** | **1** | **0** | **0** |
| Ap, Cc, Cz, Em, Km, Nx, Tm, Vm | **8.02** | **0** | **0** | **0** | **0** | **0** | **1** | **0** | **0** |
| Ap, Cc, Cz, Em, Km, Sm, Tc, Vm | **8.03** | **0** | **0** | **0** | **0** | **0** | **1** | **0** | **0** |
| Ap, Cc, Cz, Em, Km, Sm, Tm, Vm | **8.04** | **0** | **0** | **0** | **0** | **1** | **0** | **0** | **0** |
| Ap, Cc, Cz, Em, Nx, Sm, Tm, Vm | **8.05** | **0** | **0** | **0** | **0** | **0** | **0** | **0** | **43** |
| Ap, Cc, Cz, Km, Nx, Rp, Tc, Tm | **8.06** | **0** | **0** | **1** | **0** | **0** | **0** | **0** | **0** |
| Ap, Cc, Cz, Km, Nx, Sm, Tc, Tm | **8.07** | **0** | **1** | **3** | **0** | **0** | **0** | **0** | **0** |
| Ap, Cc, Cz, Km, Nx, Tc, Tm, Vm | **8.08** | **0** | **1** | **0** | **0** | **0** | **0** | **0** | **0** |
| Ap, Cc, Cz, Nx, Rp, Sm, Tc, Tm | **8.09** | **1** | **0** | **0** | **0** | **0** | **0** | **0** | **1** |
| Ap, Cc, Cz, Nx, Rp, Tc, Tm, Vm | **8.10** | **1** | **0** | **0** | **0** | **0** | **0** | **0** | **0** |
| Ap, Cc, Em, Nx, Rp, Sm, Tc, Tm | **8.11** | **0** | **0** | **0** | **1** | **0** | **0** | **0** | **0** |
| Ap, Cc, Km, Nx, Rp, Sm, Tc, Tm | **8.12** | **1** | **0** | **0** | **0** | **0** | **0** | **0** | **0** |
| Ap, Cc, Km, Nx, Sm, Tc, Tm, Vm | **8.13** | **0** | **0** | **3** | **0** | **0** | **0** | **0** | **0** |
| Ap, Cz, Em, Km, Nx, Rp, Sm, Tm | **8.14** | **0** | **0** | **0** | **0** | **0** | **0** | **1** | **0** |
| Ap, Cz, Em, Km, Nx, Sm, Tc, Tm | **8.15** | **3** | **3** | **2** | **2** | **0** | **0** | **0** | **0** |
| Ap, Cz, Em, Km, Nx, Sm, Tc, Vm | **8.16** | **0** | **0** | **0** | **0** | **0** | **1** | **0** | **0** |
| Ap, Cz, Em, Km, Rp, Sm, Tm, Vm | **8.17** | **0** | **0** | **0** | **0** | **3** | **2** | **0** | **0** |
| Ap, Cz, Em, Nx, Rp, Sm, Tc, Tm | **8.18** | **0** | **1** | **1** | **0** | **0** | **0** | **0** | **0** |
| Ap, Cz, Em, Nx, Rp, Sm, Tm, Vm | **8.19** | **0** | **0** | **0** | **0** | **1** | **1** | **0** | **0** |
| Ap, Cz, Em, Nx, Sm, Tc, Tm, Vm | **8.20** | **0** | **0** | **0** | **0** | **0** | **0** | **0** | **1** |
| Ap, Cz, Km, Nx, Rp, Sm, Tc, Tm | **8.21** | **4** | **2** | **10** | **10** | **0** | **0** | **0** | **0** |
| Ap, Cz, Km, Nx, Sm, Tc, Tm, Vm | **8.22** | **11** | **3** | **4** | **1** | **0** | **0** | **0** | **0** |
| Ap, Cc, Cz, Em, Km, Nx, Rp, Sm, Tm | **9.01** | **0** | **0** | **0** | **0** | **0** | **1** | **0** | **0** |
| Ap, Cc, Cz, Em, Km, Nx, Sm, Tc, Tm | **9.02** | **0** | **0** | **0** | **3** | **0** | **0** | **0** | **0** |
| Ap, Cc, Cz, Em, Km, Nx, Sm, Tm, Vm | **9.03** | **0** | **0** | **0** | **0** | **0** | **1** | **0** | **0** |
| Ap, Cc, Cz, Em, Nx, Rp, Sm, Tc, Tm | **9.04** | **0** | **1** | **0** | **0** | **0** | **0** | **0** | **0** |
| Ap, Cc, Cz, Em, Nx, Rp, Tc, Tm, Vm | **9.05** | **0** | **0** | **0** | **1** | **0** | **0** | **0** | **0** |
| Ap, Cc, Cz, Km, Nx, Rp, Sm, Tc, Tm | **9.06** | **1** | **0** | **2** | **0** | **0** | **0** | **0** | **0** |
| Ap, Cc, Cz, Km, Nx, Sm, Tc, Tm, Vm | **9.07** | **6** | **0** | **1** | **0** | **0** | **0** | **0** | **0** |
| Ap, Cz, Em, Km, Nx, Rp, Sm, Tc, Tm | **9.08** | **3** | **1** | **3** | **2** | **0** | **0** | **0** | **0** |
| Ap, Cz, Em, Km, Nx, Rp, Sm, Tm, Vm | **9.09** | **0** | **0** | **0** | **0** | **1** | **6** | **0** | **0** |
| Ap, Cz, Em, Km, Nx, Sm, Tc, Tm, Vm | **9.10** | **0** | **0** | **0** | **0** | **0** | **1** | **0** | **0** |
| Ap, Cz, Em, Km, Rp, Sm, Tc, Tm, Vm | **9.11** | **0** | **0** | **0** | **0** | **1** | **0** | **0** | **0** |
| Ap, Cz, Km, Nx, Rp, Sm, Tc, Tm, Vm | **9.12** | **5** | **1** | **2** | **2** | **0** | **0** | **0** | **0** |
| Ap, Cc, Cz, Em, Km, Nx, Rp, Sm, Tc, Tm | **10.01** | **0** | **1** | **1** | **0** | **0** | **0** | **0** | **0** |
| Ap, Cc, Cz, Em, Km, Nx, Rp, Sm, Tm, Vm | **10.02** | **0** | **0** | **0** | **0** | **2** | **2** | **0** | **0** |
| Ap, Cc, Cz, Em, Km, Nx, Sm, Tc, Tm, Vm | **10.03** | **0** | **0** | **0** | **0** | **0** | **0** | **2** | **0** |
| Ap, Cc, Cz, Em, Nx, Rp, Sm, Tc, Tm, Vm | **10.04** | **0** | **1** | **0** | **0** | **0** | **3** | **0** | **0** |
| Ap, Cc, Cz, Km, Nx, Rp, Sm, Tc, Tm, Vm | **10.05** | **2** | **1** | **3** | **0** | **0** | **0** | **0** | **0** |
| Ap, Cz, Em, Km, Nx, Rp, Sm, Tc, Tm, Vm | **10.06** | **1** | **1** | **0** | **1** | **0** | **0** | **0** | **0** |
| Ap, Cc, Cz, Em, Km, Nx, Rp, Sm, Tc, Tm, Vm | **11.01** | **2** | **3** | **1** | **1** | **1** | **3** | **2** | **2** |

**Table S4. MDR profiles and distribution by samples**
