# Supplementary material for: Bacteria From the Multi-Contaminated Tinto River Estuary (SW, Spain) Show High Multi-Resistance to Antibiotics and Point to Paenibacillus spp. as Antibiotic-Resistance-Dissemination Players
Source: Front Microbiol. 2020 Jan 10;10:3071. doi: 10.3389/fmicb.2019.03071 (PMC6965355; doi:10.3389/fmicb.2019.03071)
Supplement: Supplementary file 12 [file Table_5.DOCX]

| **Species (GenBank/BLAST)** | **Max. % identity** | **Max. ident. seqs. Access. no.^a^** | **No.**  **isolates** | **Species (RDP/Phylogenetic trees)^b^** |
| --- | --- | --- | --- | --- |
| *Achromobacter* sp. | 99-100 |  | 2 | *Achromobacter* *insolitus**/*spanius*/sp.* |
| *Achromobacter xylosoxidans** | 100 | AF439314 | 2 | *Achromobacter xylosoxidans*/sp.* |
| *Acinetobacter johnsonii** | 100 | KC660133 | 1 | *Acinetobacter johnsonii** |
| *Advenella incenata** | 100 | KF844056 | 1 | *Advenella* *kashmirensis* |
| *Advenella incenata*/kashmirensis* | 100 | KJ689309/HQ845175 | 1 |  |
| *Algoriphagus antarcticus* | 99 | AY771733 | 3 | *Algoriphagus* sp. |
| *Altererythrobacter dongtanensis* | 98 | NR_108695 | 1 | *Altererythrobacter* sp. |
| *Altererythrobacter ishigakiensis* | 98 | NR_112721 | 25 | *Altererythrobacter* sp. |
| *Altererythrobacter luteolus* | 100 | NR_043151 | 2 | *Altererythrobacter luteolus* |
| *Altererythrobacter xiamenensis* | 99 | NR_133694 | 1 | *Altererythrobacter xiamenensis* |
| *Arenibacter echinorum* | 99 | EF536749, KF911336 | 4 | *Arenibacter* sp. |
| *Bacillus cereus** | 98 | HM161868 | 1 | *Bacillus* sp. |
| *Bacillus cereus*/thuringiensis*/anthracis** | 100 | KP717557/ KJ676099/ KJ009486/ KP788033/ CP010106/ KP342195 | 5 | *Bacillus* *cereus*/thurigiensis*/toyonensis** |
| *Bacillus clausii* | 100 | FN397454 | 2 | *Bacillus clausii/rhizosphaerae/sp.* |
| *Bacillus galliciensis* | 99-100 | NR_116886 | 15 | *Bacillus galliciensis* |
| *Bacillus licheniformis** | 100 | HM161868, CP012110 | 3 | *Bacillus aerius*/*licheniformis** |
| *Blastomonas* sp. | 99 | FN397673 | 1 | *Erythrobacter* sp. |
| *Bosea minatitlanensis* | 99 | NR_028787 | 1 | *Bosea* *minatitlanensis/robiniae*/sp. |
| *Bosea thiooxidans* | 99 | AJ250800 | 3 | *Bosea lathyri*/sp. |
| *Bosea vestrisii** | 99 | NR_028799 | 3 | Bosea *eneae**/*vestrisii**/sp. |
| *Brachybacterium faecium* | 99 | KM186613 | 2 | *Brachybacterium* *faecium*/sp. |
| *Brachymonas chironomi* | 99 | NR_116284 | 1 | *Brachymonas chironomi* |
| *Brevundimonas bullata* | 99 | EU977700 | 1 | *Brevundimonas bullata/sp.* |
| *Brevundimonas diminuta** | 99-100 | KJ782618, KT000269 | 5 | *Brevundimonas* *naejangsanensis*/*vancanneytii** |
| *Brevundimonas* sp. | 99 |  | 1 | *Brevundimonas* sp. |
| *Brevundimonas vesicularis*/olei* | 99-100 | KM873029/NR_117268 | 5 |  |
| *Cellulosimicrobium cellulans*/funkei** | 100 | KM378605/ KM263187/ KF040973/ JQ659856/ KC429587 | 6 | *Cellulosimicrobium* *cellulans*/funkei** |
| *Chitinophaga niastensis* | 97 | AB682429 | 1 | *Chitinophaga* sp. |
| *Chitinophaga terrae*/jiangningensis* | 97 | AB682429/NR_118590 | 1 |  |
| *Citromicrobium bathyomarinum* | 99 | KF500391 | 1 | *Erythrobacter* sp. |
| *Cyclobacterium marinum* | 100 | KC534179 | 2 | *Cyclobacterium marinum* |
| *Ensifer adhaerens** | 99 | EU647697 | 1 | *Ensifer* sp. |
| *Erythrobacter aquimaris* | 99 | NR_025789 | 3 | *Erythrobacter* sp. |
| *Erythrobacter citreus* | 99-100 | GQ169076, KC534371, LC020229, KC534169 | 11 | *Erythrobacter citreus* (9)  *Erythrobacter* sp. (2) |
| *Erythrobacter gaetbuli* | 99 | FN431786 | 1 | *Erythrobacter gaetbuli* |
| *Erythrobacter gangjinensis* | 97-98 | NR_116318 | 14 | *Erythrobacter* sp. |
| *Erythrobacter longus* | 99 | AM691106 | 1 |  |
| *Erythrobacter nanhaisediminis* | 99-100 | KP342254, JQ762409 | 6 | *Erythrobacter nanhaisediminis* (1)  *Erythrobacter* sp. (5) |
| *Erythrobacter seohaensis* | 100 | NR_025817 | 2 | *Erythrobacter seohaensis* |
| *Erythrobacter* sp. | 99 | EF512713 | 2 | *Erythrobacter* sp. |
| *Erythrobacter vulgaris* | 99-100 | KF500392, KM387388, GQ169078, LK391640 | 9 | *Erythrobacter nanhaisediminis* (1)  *Erythrobacter* sp. (8) |
| *Erythrobacter vulgaris/flavus* | 99 | LK391640/EF512715 | 1 | *Erythrobacter* sp. |
| *Erythrobacter vulgaris/litoralis* | 99 | KM387388/NR_074349 | 1 |  |
| *Halomonas lionensis* | 99 | NR_126205 | 1 | *Halomonas lionensis* |
| *Joostella marina* | 100 | KF740552, KP706828 | 6 | *Joostella marina* |
| *Leeuwenhoekiella marinoflava* | 99 | KP860626 | 1 | *Leeuwenhoekiella* *marinoflava*/sp. |
| *Leucobacter komagatae* | 100 | KC492057 | 1 | *Leucobacter* sp. |
| *Lutibacterium anuloederans* | 99-100 | NR_115118 | 11 | *Erythrobacter marinus*/sp. (1)  *Erythrobacter* sp. (10) |
| *Lysinibacillus fusiformis** | 100 | KM983015, KP192023, KM983017 | 3 | *Lysinibacillus fusiformis** |
| *Lysinibacillus sphaericus** | 99 | KM378616 | 1 | *Lysinibacillus/Bacillus sphaericus** |
| *Marinobacter adhaerens/flavimaris* | 100 | KP645214/AB617558 | 2 | *Marinobacter* *adhaerens/flavimaris* |
| *Mesonia algae* | 98-100 | NR_113896 | 9 | *Mesonia algae* (8)  *Mesonia* sp. (1) |
| *Methylobacterium rhodesianum* | 100 | NR_041028, HM245434 | 16 | *Methylobacterium rhodesianum* |
| *Methylobacterium* sp. | 99 |  | 1 | *Methylobacterium* *rhodesianum*/*populi*/sp. |
| *Microbacterium lacus/aurum* | 99 | KJ812391/EU714343 | 1 | *Microbacterium* sp. |
| *Microbacterium* sp. | 97-100 |  | 18 | *Microbacterium phyllosphaera/schleiferi/oxydans** (16)  *Microbacterium* sp. (2) |
| *Mycobacterium peregrinum** | 100 | JX266704 | 1 | *Mycobacterium alvei**/*peregrinum** |
| *Neisseria* sp. | 100 | FJ502346 | 1 | *Uruburuella suis** |
| *Novosphingobium* sp./ *Altererythrobacter indicus* | 99 | AY690709/NR_043783 | 1 | *Altererythrobacter indicus* |
| *Ochrobactrum anthropi*/intermedium** | 100 | KP282813/KP259604 | 2 | *Ochrobactrum anthropi*/ciceri/intermedium** |
| *Ochrobactrum intermedium** | 100 | KM462855 | 1 |  |
| *Ochrobactrum pecoris** | 99 | NR_117053 | 2 | *Ochrobactrum pecoris** |
| *Ochrobactrum pseudogrignonense** | 100 | KF844052, KP771804 | 3 | *Ochrobactrum pseudogrignonense** |
| *Ochrobactrum pseudogrignonense*/anthropi** | 100 | KP771804/JQ612513 | 1 |  |
| *Ochrobactrum* sp. | 99-100 |  | 9 | *Ochrobactrum* *cytisi/lupini/tritici** (8)  *Ochrobactrum pecoris** (1) |
| *Paenibacillus cineris*/favisporus* | 99 | JN592451/JN867753 | 1 | *Paenibacillus* *azoreducens*/*favisporus*/sp. |
| *Paenibacillus lautus** | 99-100 | KM203759, JF798384, JF496327, JX296361 | 5 | *Paenibacillus lautus** (1)  *Paenibacillus lautus**/sp. (2)  *Paenibacillus* sp. (2) |
| *Paenibacillus* sp. | 99-100 |  | 5 | *Paenibacillus pabuli/taichungensis* (1)  *Paenibacillus* sp. (2)  *Paenibacillus tundrae*/sp. (2) |
| *Paenisporosarcina* sp. | 99 |  | 3 | *Paenisporosarcina* sp. |
| *Phycicoccus ochangensis* | 98 | GQ344407 | 3 | *Phycicoccus* sp. |
| *Ponticaulis koreensis* | 98 | NR_044608 | 14 | *Ponticaulis* sp. |
| *Pseudoalteromonas arctica* | 99 | FR750943 | 2 | *Pseudoalteromonas* sp. |
| *Pseudoalteromonas mariniglutinosa* | 98-99 | FJ457161, JQ867499 | 5 |  |
| *Pseudoalteromonas prydzensis* | 99 | KC534212 | 1 |  |
| *Pseudoalteromonas* sp. | 99-100 |  | 5 | *Pseudoalteromonas issachenkonii/tetraodonis* |
| *Pseudoalteromonas tetraodonis* | 99-100 | HM130919 | 9 | *Pseudoalteromonas* sp. |
| *Pseudomonas mendocina** | 100 | EU216597 | 1 | *Pseudomonas mendocina** |
| *Pseudomonas pseudoalcaligenes** | 100 | LK391695 | 4 | *Pseudomonas* *alcaliphila*/*oleovorans**/*pseudoalcaligenes** |
| *Pseudomonas putida** | 100 | KR054999 | 1 | *Pseudomonas* *fulva**/*mosselii**/*putida**/sp. |
| *Pseudomonas* sp. | 100 |  | 1 | *Pseudomonas fulva** |
| *Pseudomonas stutzeri** | 100 | AJ312161 | 1 | *Pseudomonas xanthomarina* |
| *Salegentibacter mishustinae* | 99 | KF500388 | 1 | *Salegentibacter mishustinae* |
| *Salinimicrobium* sp. | 100 | FJ425217 | 1 | *Salinimicrobium* *terrae*/sp. |
| *Solibacillus silvestris* | 99 | LN870317 | 1 | *Solibacillus* *isronensis*/*silvestris* |
| *Sphingobacterium detergens* | 99 | NR_118238 | 2 | *Sphingobacterium* *detergens*/sp. |
| *Sphingobacterium hotanense** | 97-99 | NR_108440 | 3 | *Sphingobacterium hotanense** (2)  *Sphingobacterium* sp. (1) |
| *Sphingobacterium* sp. | 97 | NR_074508 | 1 | *Olivibacter* sp. |
| *Sphingobium xenophagum* | 99 | KJ676967 | 1 | *Sphingobium* *xenophagum*/sp. |
| *Sphingopyxis* sp. | 100 |  | 3 | *Sphingopyxis* sp. |
| *Sphingopyxis flavimaris* | 99 | NR_025814 | 1 | *Sphingorhabdus flavimaris* |
| *Sphingopyxis ginsengisoli* | 100 | JQ342928 | 1 | *Sphingopyxis ginsengisoli* |
| *Sphingopyxis litoris* | 99 | KC756877, DQ781321 | 5 | *Sphingorhabdus* *litoris*/sp. (3)  *Sphingorhabdus* sp. (3) |
| *Sphingopyxis macrogoltabida* | 99 | AB675371 | 1 | *Sphingopyxis* sp. |
| *Sphingomonas azotofornans* | 98 | AB681850 | 2 | *Sphingobium* sp. |
| *Stenotrophomonas maltophilia** | 100 | KR083021, KP185140, KP986951, JX262398 | 4 | *Stenotrophomonas maltophilia** (1)  *Stenotrophomonas* *maltophilia*/*sp. (1)  *Stenotrophomonas maltophilia*/pavanii** (1)  *Stenotrophomonas* sp. (1) |
| *Streptomyces indicus* | 99 | NR_108182 | 1 | *Streptomyces indicus* |
| *Streptomyces* sp. | 100 |  | 1 | *Streptomyces* *africanus*/*mutabilis*/*rochei* |
| *Streptomyces xiamenensis* | 100 | CP009922 | 1 | *Streptomyces xiamenensis* |
| *Tenacibaculum gallaicum** | 99 | NR_042631 | 4 | *Tenacibaculum gallaicum** (2)  *Tenacibaculum* *gallaicum*/sp.* (2) |
| *Tsukamurella inchonensis** | 100 | AB907635 | 1 | *Tsukamurella* *spumae*/inchonensis** |
| *Virgibacillus carmonensis* | 99 | NR_025481 | 1 | *Virgibacillus/Oceanobacillus* sp. |
| *Zunongwangia profunda* | 99 | LN651154 | 1 | *Zunongwangia* *profunda*/sp. |

**Table S5. Phylogenetic ascription of isolates by two methods based on 16S rRNA sequences. For ascriptions based on BLAST analysis genus name is followed by sp. when maximum identity is found for an unidentified species o more of two show the same percentage of identity. ^a^Accession numbers of GenBank sequences with the highest similarity are shown. When many sequences give the same percentage of identity no accession number is shown. ^b^Number of isolates with the same ascription in the analysis performed based on phylogenetic trees built using type strain sequences at RDP are shown in brackets. When an isolate shares position in the tree with several species or is not close to any, sp. is used after the genus name. * Indicates bacterial species or genus for which infections on humans, animals or plants have been reported.**
