# Supplementary material for: Bacteria From the Multi-Contaminated Tinto River Estuary (SW, Spain) Show High Multi-Resistance to Antibiotics and Point to Paenibacillus spp. as Antibiotic-Resistance-Dissemination Players
Source: Front Microbiol. 2020 Jan 10;10:3071. doi: 10.3389/fmicb.2019.03071 (PMC6965355; doi:10.3389/fmicb.2019.03071)
Supplement: Supplementary file 13 [file Table_6.DOCX]

|  | **All isolates** | **Actinobacteria** | **α-proteobacteria** | **Bacilli** | **β-proteobacteria** | **Flavobacteriia** | **γ-proteobacteria** | **Sphingobacteriia** | **Altererythrobacter** | **Bacillus** | **Brevundimonas** | **Erythrobacter** | **Mesonia** | **Microbacterium** | **Ochrobactrum** | **Paenibacillus** | **Ponticaulis** | **Pseudoalteromonas** | **Pseudomonas** |
| --- | --- | --- | --- | --- | --- | --- | --- | --- | --- | --- | --- | --- | --- | --- | --- | --- | --- | --- | --- |
| **Cc/Cz** | **0.076** | **0.000** | **0.073** | **0.021** | **0.750** | **0.032** | **0.156** |  | **0.000** | **0.033** |  | **0.028** | **0.372** | **0.198** |  | **0.290** | **0.041** | **0.172** | **0.250** |
| **Cc/Em** | **0.309** | **0.206** | **0.237** | **0.424** |  | **0.000** | **0.631** | **0.385** | **0.000** | **0.091** | **0.154** | **0.010** | **0.000** | **0.357** |  | **0.814** | **0.632** | **0.351** | **1.000** |
| **Cc/Km** | **0.155** | **0.029** | **0.106** | **0.474** | **0.250** |  | **0.103** | **0.000** | **0.000** | **0.091** | **0.233** | **0.067** | **0.609** | **0.222** | **0.000** | **0.814** | **0.186** | **0.211** | **0.250** |
| **Cc/Nx** | **0.103** | **0.153** | **0.039** | **0.122** |  |  | **0.464** | **0.467** | **0.000** | **0.033** |  |  | **0.609** | **0.250** | **0.571** | **0.633** | **0.109** | **0.411** | **0.750** |
| **Cc/Rp** | **0.204** | **0.323** | **0.060** | **0.424** | **0.500** | **0.320** | **0.362** | **0.158** | **0.000** | **0.455** | **0.279** | **0.063** | **0.250** | **0.852** | **0.143** | **0.421** | **0.632** | **0.488** | **0.250** |
| **Cc/Sm** | **0.006** | **0.183** |  | **0.122** |  |  | **0.015** | **0.385** |  | **0.072** |  |  |  | **0.088** |  | **0.127** | **0.025** | **0.075** | **0.000** |
| **Cc/Tc** | **0.052** | **0.091** | **0.077** | **0.188** | **0.750** | **0.129** | **0.056** | **1.000** | **0.000** | **0.057** | **0.279** |  | **1.000** | **0.063** | **0.143** | **0.459** |  | **0.105** | **0.250** |
| **Cc/Tm** | **0.076** | **0.064** | **0.053** | **0.017** | **0.500** | **0.224** | **0.100** | **0.143** |  |  | **0.094** | **0.067** | **0.609** | **0.063** |  | **0.290** | **0.109** | **0.075** | **0.250** |
| **Cc/Vm** | **0.316** | **0.495** | **0.285** | **0.346** | **0.250** | **0.023** | **0.015** | **0.750** | **0.000** | **0.000** | **0.222** | **0.315** | **0.250** | **0.609** |  | **0.214** | **0.243** | **0.033** | **0.250** |
| **Cz/Em** | **0.148** | **0.159** | **0.169** | **0.154** |  | **0.000** | **0.180** |  | **0.164** | **0.108** |  | **0.123** | **0.000** | **0.198** |  | **0.492** | **0.087** | **0.039** | **0.250** |
| **Cz/Km** | **0.297** | **0.324** | **0.228** | **0.128** | **0.529** |  | **0.668** |  | **0.273** | **0.054** |  | **0.333** | **0.182** | **0.333** |  | **0.492** | **0.075** | **0.667** | **0.385** |
| **Cz/Nx** | **0.187** | **0.340** | **0.091** | **0.322** |  |  | **0.510** |  | **0.416** | **0.286** |  |  | **0.182** | **0.348** |  | **0.645** | **0.222** | **0.347** | **0.467** |
| **Cz/Rp** | **0.125** | **0.302** | **0.012** | **0.154** | **0.714** | **0.381** | **0.203** |  | **0.190** | **0.108** |  | **0.091** | **0.769** | **0.258** |  | **0.225** | **0.116** | **0.125** | **0.385** |
| **Cz/Sm** | **0.208** | **0.265** |  | **0.445** |  |  | **0.490** |  |  | **0.113** |  |  |  | **0.014** |  | **0.792** | **0.759** | **0.588** | **0.143** |
| **Cz/Tc** | **0.061** | **0.107** | **0.098** | **0.088** | **1.000** | **0.235** | **0.288** |  | **0.295** | **0.091** |  |  | **0.372** | **0.087** |  | **0.441** |  | **0.222** | **0.385** |
| **Cz/Tm** | **0.358** | **0.340** | **0.320** | **0.486** | **0.714** | **0.295** | **0.391** |  |  |  |  | **0.308** | **0.182** | **0.348** |  | **1.000** | **0.533** | **0.588** | **0.059** |
| **Cz/Vm** | **0.155** | **0.018** | **0.221** | **0.042** | **0.385** | **0.366** | **0.234** |  | **0.044** | **0.000** |  | **0.183** | **0.308** | **0.092** |  | **0.108** | **0.263** | **0.481** | **0.059** |
| **Em/Km** | **0.137** | **0.238** | **0.161** | **0.513** |  |  | **0.110** |  | **0.173** | **0.600** | **0.055** | **0.167** | **0.000** | **0.444** | **0.000** | **1.000** | **0.045** | **0.049** | **0.250** |
| **Em/Nx** | **0.048** | **0.134** | **0.113** | **0.179** |  |  | **0.465** | **0.385** | **0.120** | **0.054** |  |  | **0.000** | **0.250** | **0.000** | **0.814** | **0.037** | **0.104** | **0.750** |
| **Em/Rp** | **0.245** | **0.267** | **0.191** | **0.328** |  | **0.000** | **0.454** | **0.040** | **0.119** | **0.200** | **0.055** | **0.094** | **0.000** | **0.262** | **0.000** | **0.560** | **0.417** | **0.462** | **0.250** |
| **Em/Sm** | **0.087** | **0.274** |  | **0.179** |  |  | **0.029** | **1.000** |  | **0.032** |  |  |  | **0.308** |  | **0.353** | **0.054** | **0.017** | **0.000** |
| **Em/Tc** | **0.188** | **0.018** | **0.382** | **0.262** |  | **0.000** | **0.110** | **0.385** | **0.307** | **0.000** | **0.055** |  | **0.000** | **0.125** | **0.000** | **0.645** |  | **0.023** | **0.250** |
| **Em/Tm** | **0.084** | **0.047** | **0.104** | **0.118** |  | **0.000** | **0.067** | **0.600** |  |  | **0.233** | **0.058** | **0.000** | **0.063** |  | **0.492** | **0.222** | **0.017** | **0.250** |
| **Em/Vm** | **0.215** | **0.103** | **0.327** | **0.089** |  | **0.000** | **0.029** | **0.250** | **0.194** | **0.000** | **0.128** | **0.191** | **0.000** | **0.217** |  | **0.298** | **0.103** | **0.023** | **0.250** |
| **Km/Nx** | **0.154** | **0.244** | **0.081** | **0.235** |  |  | **0.512** |  | **0.264** | **0.108** |  |  | **0.125** | **0.222** | **0.273** | **0.814** | **0.440** | **0.438** | **0.385** |
| **Km/Rp** | **0.173** | **0.309** | **0.168** | **0.235** | **0.333** |  | **0.222** |  | **0.012** | **0.200** | **0.375** | **0.133** | **0.118** | **0.333** | **0.207** | **0.560** | **0.364** | **0.154** | **0.143** |
| **Km/Sm** | **0.116** | **0.116** |  | **0.150** |  |  | **0.436** |  |  | **0.032** |  |  |  | **0.111** |  | **0.353** | **0.222** | **0.500** | **0.200** |
| **Km/Tc** | **0.291** | **0.324** | **0.172** | **0.316** | **0.529** |  | **0.446** |  | **0.207** | **0.182** | **0.083** |  | **0.609** | **0.222** | **0.116** | **0.645** |  | **0.400** | **0.143** |
| **Km/Tm** | **0.159** | **0.244** | **0.140** | **0.103** | **0.333** |  | **0.371** |  |  |  | **0.128** | **0.300** | **0.125** | **0.222** |  | **0.492** | **0.120** | **0.500** | **0.158** |
| **Km/Vm** | **0.232** | **0.136** | **0.150** | **0.305** | **0.158** |  | **0.323** |  | **0.089** | **0.000** | **0.233** | **0.100** | **0.118** | **0.000** |  | **0.298** | **0.553** | **0.400** | **0.158** |
| **Nx/Rp** | **0.158** | **0.359** | **0.023** | **0.266** |  |  | **0.360** | **0.158** | **0.221** | **0.108** |  |  | **0.118** | **0.323** | **0.222** | **0.421** | **0.222** | **0.310** | **0.385** |
| **Nx/Sm** | **0.350** | **0.320** |  | **0.639** |  |  | **0.178** | **0.385** |  | **0.620** |  |  |  | **0.400** |  | **0.476** | **0.364** | **0.079** | **0.143** |
| **Nx/Tc** | **0.338** | **0.528** | **0.260** | **0.494** |  |  | **0.292** | **0.467** | **0.454** | **0.727** |  |  | **0.609** | **0.500** | **0.075** | **0.459** |  | **0.352** | **0.385** |
| **Nx/Tm** | **0.301** | **0.397** | **0.198** | **0.441** |  |  | **0.339** | **0.714** |  |  |  |  | **1.000** | **0.250** |  | **0.645** | **0.417** | **0.263** | **0.529** |
| **Nx/Vm** | **0.028** | **0.134** | **0.023** | **0.047** |  |  | **0.178** | **0.250** | **0.063** | **0.000** |  |  | **0.118** | **0.118** |  | **0.214** | **0.588** | **0.167** | **0.529** |
| **Rp/Sm** | **0.116** | **0.307** |  | **0.266** | **0.000** |  | **0.102** | **0.040** |  | **0.226** |  |  |  | **0.011** |  | **0.154** | **0.054** | **0.054** | **0.200** |
| **Rp/Tc** | **0.110** | **0.302** | **0.052** | **0.355** | **0.714** | **0.129** | **0.203** | **0.158** | **0.390** | **0.182** | **0.375** |  | **0.250** | **0.129** | **0.138** | **0.313** |  | **0.075** | **1.000** |
| **Rp/Tm** | **0.165** | **0.243** | **0.168** | **0.118** | **0.333** | **0.224** | **0.117** | **0.091** |  |  | **0.154** | **0.142** | **0.118** | **0.129** |  | **0.225** | **0.037** | **0.054** | **0.158** |
| **Rp/Vm** | **0.145** | **0.267** | **0.046** | **0.271** | **0.600** | **0.324** | **0.102** | **0.250** | **0.175** | **0.000** | **0.279** | **0.063** | **0.500** | **0.491** |  | **0.621** | **0.103** | **0.057** | **0.158** |
| **Sm/Tc** | **0.145** | **0.029** |  | **0.268** |  |  | **0.490** | **0.385** |  | **0.400** |  |  |  | **0.080** |  | **0.241** |  | **0.488** | **0.200** |
| **Sm/Tm** | **0.203** | **0.168** |  | **0.301** |  |  | **0.421** | **0.600** |  |  |  |  |  | **0.160** |  | **0.792** | **0.364** | **0.611** | **0.111** |
| **Sm/Vm** | **0.033** | **0.113** |  | **0.047** |  |  | **0.266** | **0.250** |  | **0.000** |  |  |  | **0.042** |  | **0.072** | **0.169** | **0.488** | **0.111** |
| **Tc/Tm** | **0.151** | **0.340** | **0.037** | **0.329** | **0.714** | **0.416** | **0.391** | **0.143** |  |  | **0.154** |  | **0.609** | **0.250** |  | **0.441** |  | **0.488** | **0.158** |
| **Tc/Vm** | **0.011** | **0.159** | **0.195** | **0.065** | **0.385** | **0.264** | **0.362** | **0.750** | **0.128** | **0.000** | **0.744** |  | **0.250** | **0.118** |  | **0.154** |  | **0.382** | **0.158** |
| **Tm/Vm** | **0.127** | **0.047** | **0.105** | **0.019** | **0.600** | **0.273** | **0.586** | **0.000** |  |  | **0.094** | **0.058** | **0.118** | **0.059** |  | **0.108** | **0.314** | **0.829** | **0.467** |

**Table S6. Kappa (κ) index positive values for pairs of antibiotic resistances in specific groups of isolates. κ>0.4 are highlighted with grey background. Empty cells correspond to zero or negative values or because one or the two resistances of the pair was considered putative inR for that group of isolates.**
